# Down the membrane hole: Ion channels in protozoan parasites

**DOI:** 10.1371/journal.ppat.1011004

**Published:** 2022-12-29

**Authors:** Veronica Jimenez, Sebastian Mesones

**Affiliations:** Department of Biological Science, College of Natural Sciences and Mathematics, California State University Fullerton, Fullerton, California, United States of America; Joan and Sanford I Weill Medical College of Cornell University, UNITED STATES

## Abstract

Parasitic diseases caused by protozoans are highly prevalent around the world, disproportionally affecting developing countries, where coinfection with other microorganisms is common. Control and treatment of parasitic infections are constrained by the lack of specific and effective drugs, plus the rapid emergence of resistance. Ion channels are main drug targets for numerous diseases, but their potential against protozoan parasites is still untapped. Ion channels are membrane proteins expressed in all types of cells, allowing for the flow of ions between compartments, and regulating cellular functions such as membrane potential, excitability, volume, signaling, and death. Channels and transporters reside at the interface between parasites and their hosts, controlling nutrient uptake, viability, replication, and infectivity. To understand how ion channels control protozoan parasites fate and to evaluate their suitability for therapeutics, we must deepen our knowledge of their structure, function, and modulation. However, methodological approaches commonly used in mammalian cells have proven difficult to apply in protozoans. This review focuses on ion channels described in protozoan parasites of clinical relevance, mainly apicomplexans and trypanosomatids, highlighting proteins for which molecular and functional evidence has been correlated with their physiological functions.

## Introduction

The establishment of lipidic boundaries in cells is a key evolutionary event that defines the existence of two distinct compartments, the intracellular and the extracellular space. The asymmetric distribution of molecules across the plasma membrane creates an electrochemical gradient that all living organisms use as energy source for the exchange of nutrients, water, and metabolites. Flow between compartments is mediated by ion channels and transporters, transmembrane proteins selectively carrying molecules across the lipid barrier. By controlling the flow of ions, channels regulate a diverse array of cellular functions including cell volume, motility, signaling, excitability, and cell death [1–7].

Phylogenetic analyses shows that ion channels are present in all prokaryotic and eukaryotic cells, with the common ancestors derived from bacterial porins and ABC transporters [8]. The type and abundance of channels and transporters often respond to the needs of each organism to cope with environmental conditions. Thus, the set of membrane proteins expressed by a cell equips them with the necessary tools to perform their function, for example, excitable cells expressed a higher number and diversity of ion channels compared with nonexcitable cells [9]. Structurally and functionally diverse, ion channels possess conserved core properties associated with the pore segments, and through their evolutionary history, they have acquired modular domains that regulate their activity and provide responsive elements linking the channels’ gating with cell signaling events [10–13].

Traditionally, channels were classified based on their selective permeability to the most abundant ions in the intracellular and extracellular environments. As such, sodium, potassium, proton, chloride, and calcium channels cover most of the exchange between cells and their surroundings. Channels can be also classified by their gating or activation mode, depending on their response to changes in voltage, binding of intracellular or extracellular ligands, or interaction with the membrane lipids. In the past 20 years, the increasing knowledge on the role of channels in sensing modalities gave origin to a new classification based on their ability to detect oxygen, acid, mechanical stimuli, temperature, etc. [14]. These types of channels are particularly interesting in the context of host–parasite interactions, as they could be involved in the detection of environmental conditions that regulate developmental transitions.

The first crystallographic structure of a potassium channel was obtained in 1998 when the small bacterial KcsA channel was described [15]. Since then, hundreds of channels have been characterized in bacteria, fungi, plants, and mammals [14,16,17], and their roles in the most diverse physiological functions from survival to death have been established. In contrast, only a few channels have been identified or even partially characterized in protozoan parasites (Tables 1 and 2). Despite the relevance of ionic control in protozoans, our knowledge of the specific molecules responsible for the permeability of ions across the plasma membrane and the intracellular organelles is still limited and fragmentary. Low sequence homology with known channels, lack of response to selective blockers, and technical difficulties of direct electrophysiological recordings in small motile cells are some of the challenges that limit our knowledge of ion channel physiology in these parasites [18]. In the following sections, we will summarize current information describing the physiological role of ion channels as regulators of cellular functions and host–parasite interaction. We will describe channels residing in the plasma membrane and main intracellular organelles, since channel localization and abundance is a determinant of ion exchange between compartments. We will dedicate a special section to proteins produced by the parasites and exported to the membranes of host cells where they mediate nutrient exchange. Finally, we will propose future research directions to increase our understanding of ion physiology in unicellular parasites while offering insights on their potential as selective drug targets.

**Table 1:** Ion channels in pathogenic trypanosomatids.

Name	Gene ID	Organism	Localization	Reference
TcCat	TcCLB.507213.30	*Trypanosoma cruzi*	Plasma membrane	[76]
TcCAKC	TcCLB.506529.150	*Trypanosoma cruzi*	Plasma membrane	[82]
TcMscS	TcCLB.504171.40	*Trypanosoma cruzi*	Contractile vacuole-plasma membrane	[111]
Tc Aquaporin 1		*Trypanosoma cruzi*	Acidocalcisomes, Contractile vacuole	[135,136]
Calcium channel (putative)	TcCLB.504105.130	*Trypanosoma cruzi*	Contractile vacuole-plasma membrane	[110,333]
TcIP3R	TcCLB.509461.90	*Trypanosoma cruzi*	Acidocalcisomes	[214,334]
TcMCU	TcCLB.503893.120	*Trypanosoma cruzi*	Mitochondria	[207,208]
TbMCU	Tb927.11.1350	*Trypanosoma brucei*	Mitochondria	[205,206]
TbIP3R	Tb927.8.2770	*Trypanosoma brucei*	Acidocalcisomes	[215]
Aquaglyceroporin 1	Tb927.6.1520	*Trypanosoma brucei*	Flagellum	[138,335]
Aquaglyceroporin 2	Tb927.10.14170	*Trypanosoma brucei*	Flagellar pocket	[138,140,335]
Aquaglyceroporin 3	Tb927.10.14160	*Trypanosoma brucei*	Plasma membrane	[138,335]
VDAC1	Tb927.2.2510	*Trypanosoma brucei*	Mitochondria	[197]
VDAC2	Tb927.2.2520	*Trypanosoma brucei*	Mitochondria	[200]
TbK1	Tb927.1.4450	*Trypanosoma brucei*	Plasma membrane	[83]
TbK2	Tb927.9.4820	*Trypanosoma brucei*	Plasma membrane	[83]
TbIRK	Tb927.11.12490	*Trypanosoma brucei*	Acidocalcisomes	[246]
TbVCL1	Tb927.9.8540	*Trypanosoma brucei*	Golgi apparatus	[336]
TbVCL2	Tb927.10.11680	*Trypanosoma brucei*	Endoplasmic reticulum	[336]
TbVCL3	Tb927.11.16690	*Trypanosoma brucei*	Endoplasmic reticulum	[336]
Calcium channel (putative)	Tb927.10.2880	*Trypanosoma brucei*	Flagellum	[114]
LmAQP1	LmjF.31.0020	*Leishmania major*	Flagellum, flagellar pocket, contractile vacuole	[131,132]
Calcium channel (putative)	LmxM.17.1440	*Leishmania mexicana*	Plasma membrane	[105]
Calcium channel (putative)	LmxM.33.0480	*Leishmania mexicana*	Plasma membrane	[105]

**Table 2:** Ion channels in apicomplexans and other pathogenic protozoans.

Name	Gene ID	Organism	Localization	Reference
TgAQP1	TGME49_215450	*Toxoplasma gondii*	Plant-like vacuole	[128,223,337]
TgTPC	TGGT1_311080	*Toxoplasma gondii*	Apicoplast	[57]
TgTRPPL-2	TgGT1_310560	*Toxoplasma gondii*	Plasma membrane, ER	[52]
TgVDAC	TGME49_263300	*Toxoplasma gondii*	Mitochondria	[195]
PSAC- Clag3.1	PF3D7_0302500	*Plasmodium falciparum*	Erythrocyte plasma membrane	[265]
PSAC- Clag3.2	PF3D7_0302200	*Plasmodium falciparum*	Erythrocyte plasma membrane	[265]
PfAQP	PF3D7_1132800	*Plasmodium falciparum*	Plasma membrane	[129,338]
PbAQP	PBANKA_0915600	*Plasmodium berghei*	Plasma membrane	[339,340]
PfKch1	PF3D7_1227200	*Plasmodium falciparum*	Erythrocyte plasma membrane	[87,341]
PfKch2	PF3D7_1465500	*Plasmodium falciparum*	Plasma membrane	[23,87]
PbKch1	PBANKA_1442000	*Plasmodium berghei*	Plasma membrane?	[90]
PbKch2	PBANKA_1328900	*Plasmodium berghei*	Plasma membrane?	[89]
Amoebapores		*Entamoeba histolytica*	Exported	[342–346]
EhClC-A	EHI_142170	*Entamoeba histolytica*	Plasma membrane	[121]
CLC2	ND	*Giardia intestinalis*	Plasma membrane	[122]

## Physiological roles of ion channels in protozoans

The ionic composition of the cytosol and organelles in protozoan parasites is similar to that of mammalian cells, with K^+^ as the predominant intracellular cation, Ca^2+^ concentrations around 100 nM and variable amounts of Cl^−^ and organic anions [19]. Consequently, we must assume the presence of ion channels and transporters sustaining the electrochemical gradients and the membrane potential. Genome-wide and phenotypic analyses conducted in *Plasmodium* showed a reduced number of channels and transporters expressed in the parasites compared with bacteria and mammalian cells [20–22], suggesting the selection of a minimum transportome that supports the survival and replication of protozoans adapted to a parasitic lifestyle [23–26]. The lack of redundancy on the transport mechanisms also suggests the essentiality of these proteins, but conclusive functional evidence is needed to demonstrate this hypothesis.

**Fig 1:**
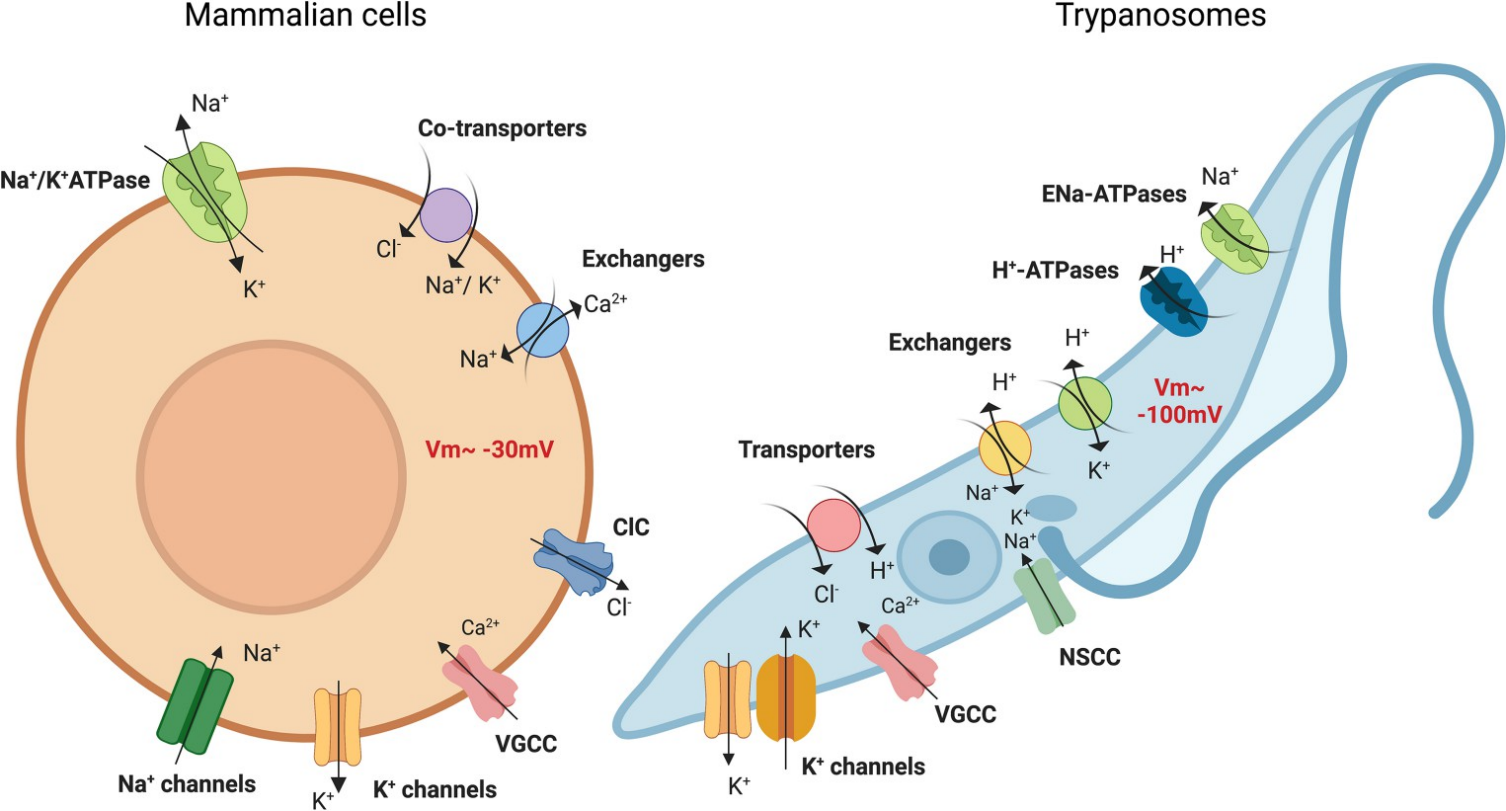
**Channels and transporters involved in maintenance of the membrane potential in nonexcitable mammalian cells (A) and trypanosomes (B).** In nonexcitable mammalian cells, the resting membrane potential (Vm) is maintained at values close to −30 mV by the coordinated activity of ion channels and transporters. This membrane potential is driven by a high permeability to K^+^ caused by the abundant expression of leak K^+^ channels. Na^+^ influx via channels, exchangers, and cotransporters is counterbalanced by the activity of the Na^+^/K^+^ ATPase, main membrane pump found in vertebrates. Cl^−^, the most abundant intracellular anion, is regulated by electroneutral transporters such as the Cl^−^-Na^+^-K^+^ cotransporter and by ClC channels. While Ca^2+^ influx via VGCCs does not cause a significant change in the membrane potential directly, the cascade of signaling events associated with Ca^2+^ entry affects expression and trafficking of the channels and transporters mentioned above. In trypanosomatids, and other protozoan parasites studied to date, membrane potential is driven by a proton motive force generated by the activity of H^+^ ATPases, establishing a value of about −100 mV for *T*. *cruzi* epimastigotes. Depending on the life stages analyzed, K^+^ plays a secondary role on Vm maintenance and the hyperpolarization supports the influx of K^+^ by channels and/or via exchangers. No Na^+^ channels have been identified but permeation occurs through NSCCs and exchangers, while efflux is mediated by ENa ATPases. *Figure created with BioRender.com*. NSCC, nonselective cation channel; VGCC, voltage-gated Ca^2+^ channel.

The Na^+^/K^+^ ATPase is the predominant pump operating in the plasma membrane of mammalian cells [27,28], but its identification in unicellular parasites has been elusive, with some reports of its function in *Leishmania* [29]. Like plants, protozoans abundantly express H^+^-ATPases and regulate most of their membrane processes based on H^+^ motive force instead of Na^+^ or K^+^ fueled transport. Therefore, proton-dependent membrane potential and pH regulation are strictly linked in the parasites, while other monovalent cations are required to support the activity of pumps and transporters that control nutrient exchange (Fig 1).

The membrane potential (Vm) of multiple protozoans has been measured and is generally more negative than in nonexcitable mammalian cells, with values averaging −80 to −120 mV [30–35]. Multiple studies have confirmed the H^+^ dependency of the membrane potential and a relative insensitivity to the presence of monovalent cations in the media, with some exceptions. *T*. *brucei* bloodstream trypomastigotes and *P*. *falciparum* trophozoites are strongly depolarized in high K^+^ media [30,35] and hyperpolarized in the presence of Ba^2+^, suggesting the presence of a channel mediating the influx of K^+^ to counterbalance the activity of the membrane H^+^-ATPases. Chloride conductance also influences membrane potential, and it has been postulated that an anion channel may be responsible for the Cl^−^ uptake and concomitant depolarization observed in *Leishmania* [32] and *Plasmodium* [36]. The hyperpolarized Vm observed in protozoan parasites compared with mammalian cells may provide buffering capacity for H^+^ mobilization, necessary to maintain tight control of the intracellular pH, as it has been shown in bacteria [16,37]. Remarkably, protozoans are able to keep their intracellular pH constant under challenging external conditions such as the incorporation into phagolysosomes [38,39] or the transit through the intestinal track of insect vectors [40]. Furthermore, cues such as media acidification are used as signals to trigger their differentiation [41,42] and to modify their behavior [43]. Upon variations in extracellular pH the Vm of trypanosomatids varies, becoming more depolarized under alkaline conditions and hyperpolarized at acidic pH. These changes are quickly compensated by the mobilization of H^+^ via H^+^-ATPases [30,31,44,45]. Thus, changes in pH can be transduced intracellularly as fluctuations in membrane potential, triggering downstream effects. For example, plasma membrane depolarization activates voltage-gated channels causing changes in cytosolic Ca^2+^ [46,47]. This is, perhaps, the most relevant signaling event in parasite’s physiology since Ca^2+^ oscillations control gene expression, differentiation, motility, invasion, and egress from host cells [4,48–55]. Calcium signaling also determines the fate of cells, stimulating replication and triggering cell death [56–61]. While essential cellular functions are strongly dependent and tightly controlled by ionic conditions, our understanding of the molecules that regulate ion exchange in the parasites is limited. In the following sections, we will summarize the current knowledge of ion channels in protozoans focusing on proteins that have been molecularly identified and functionally analyzed. For clarity, we will use the localization of the channels as their primary classification criteria, with some exceptions due to the complexity of the topic.

## Ion channel repertoires

### Plasma membrane channels

The separation of the intracellular and extracellular environments by a selectively permeable lipid barrier implies the need for transmembrane proteins facilitating the exchange of ions and other molecules. Na^+^ and K^+^ gradients, together with Ca^2+^ oscillations, are major stimuli that regulate cell volume, cell survival, and signaling [2]. The predominance of Na^+^ in blood and tissue fluids and the intracellular acidification observed in the absence of extracellular Na^+^ [62–64] suggest the presence of Na^+^ channels in the plasma membrane of extracellular parasites, but their molecular identity is still obscure. Curiously, ouabain-sensitive Na^+^/K^+^ ATPases, extremely abundant in mammalian cells, do not seem to play a significant role in ion homeostasis in protozoans; instead, Na^+^ pumps are ubiquitously expressed. In *Plasmodium falciparum* PfATP4, initially described as a Ca^2+^ pump [65], was later demonstrated to be a plasma membrane Na^+^ efflux pump [66,67] associated with resistance to antimalarials [68,69]. TgATP4, a homolog found in *Toxoplasma gondii*, controls the cytosolic concentration of Na^+^ [70]. In *Trypanosoma cruzi*, a Na^+^ ATPase insensitive to ouabain (TcENA) is more abundantly expressed in epimastigotes and trypomastigotes compared with intracellular amastigotes [71,72]. *Trypanosoma brucei* and *Leishmania* [73] also possess P-type Na^+^ ATPases similar to TcENA [74,75]. The primary role of ENA ATPases is to maintain a Na^+^ gradient that fuels secondary transport of nutrients, and their function is necessary linked to the influx of Na^+^ via ion channels. Despite the indirect evidence of their presence, not a single Na^+^ selective channel has been identified in protozoan parasites. It is possible that nonselective cation channels such as TcCat [76] serve as permeation pathways for both, Na^+^ and K^+^, depending on the electrochemical gradients, thus fulfilling the role of Na^+^ channels, but the complete absence of this type of channels is unlikely and further research exploring this question is needed.

While in mammalian cells K^+^ permeability is a major contributor to membrane potential maintenance [77], this ion plays a secondary role in protozoans, with proton-motive forces driving most of the resting membrane potential [30,31,33–35,78]. K^+^, as the most abundant cytosolic cation, is essential for the function of H^+^/K^+^ exchangers, and its concentration has a profound effect on the regulation of cell volume, enzymatic activity, and cell death [1,77]. Analysis of protozoan genomes predicts the presence of sequences encoding for voltage and calcium-gated potassium channels as well as inward rectifiers [79], and a discrete number of channels have been cloned and characterized (Tables 1 and 2). Electrophysiological recordings of proteoliposomes containing membranes isolated from *T*. *cruzi* epimastigotes showed the presence of at least two distinct potassium pathways: a larger channel with a conductance of around 100 pS, mostly permeable to K^+^, and a nonselective cation channel, with lower conductance and slight rectification [80]. This channel shares some functional characteristics with TcCat, a nonselective cation channel expressed in all life stages of *T*. *cruzi* [76], including blockage by divalent cations and similar conductance, but they differ in their selectivity and permeability sequence. These functional differences may indicate the presence of two different nonselective cation channels or could correspond to the same channel whose activity is modulated by the lipidic environment and or/signaling, often lost when the proteins are expressed in heterologous systems. The nonselective nature of TcCat agrees with the absence of a conserved selectivity filter, a feature found in all K^+^ selective channels [15,81]. The channel localizes to the plasma membrane of the parasites and is required for volume regulation under hyperosmotic conditions [76], a function often dependent on the mobilization of K^+^. A second K^+^ channel was described in *T*. *cruzi* and showed features of a calcium-activated K^+^ channel (TcCAKC). This protein plays a central role in regulating intracellular pH and membrane potential in *T*. *cruzi* [82] and its ablation in epimastigotes induced hyperpolarization and cytosolic alkalinization, as well as an increase in the rate of proton extrusion. Importantly, TcCAKC knockout parasites were unable to differentiate into trypomastigotes, severely impairing their infectivity and emphasizing the role of K^+^ homeostasis in parasite fitness. Previously, it has been shown that trypomastigotes membrane potential is more sensitive to extracellular K^+^ than that of epimastigotes [31], and this is also the case for *T*. *brucei* [30]. TbK1 and TbK2 form heterodimeric K^+^ channels required for membrane potential regulation and cell survival in *T*. *brucei* bloodstream forms, while being dispensable in procyclic forms. These results suggest a tighter control of K^+^ conductance at the plasma membrane in the bloodstream stages [83] in vitro but raise the question of whether this ion could have a more drastic influence on parasites survival when evaluated in vivo. Indirect evidences suggest the presence of calcium-activated K^+^ channels [84] and nonselective cation channels in *Leishmania* amastigotes and promastigotes [85,86], but no molecular or biophysical characterization of these putative proteins has been reported.

In apicomplexans, a limited number of plasma membrane channels have been characterized (Table 2). PfKch1 is a six-transmembrane domain protein with a conserved selectivity filter sequence that is expressed throughout the erythrocytic cycle, with higher expression in trophozoites and schizonts [87]. Interestingly, as the parasites mature, the protein could be detected in the plasma membrane of infected red blood cells (RBCs), suggesting the export of the channel during later developmental stages. PfKch2 is a smaller protein, found in the plasma membrane of schizonts and merozoites [87]. Attempts to knock out these channels have failed and exposure to calcium-activated K^+^ channel blockers like clotrimazole had a potent antimalarial effect, suggesting the essentiality of PfKch1 and PfKch2. Recently, expression of these two proteins in *Saccharomyces cerevisiae* showed their membrane localization and ability to form functional channels when reconstituted in lipid bilayers [88]. Rb^+^ uptake experiments in *Plasmodium berghei* validate the role of the homolog PbKch1 and PbKch2 as K^+^ permeation pathways. While PbKch2 knockout did not have a drastic effect in asexual or sexual developmental stages [89], PbKch1-null parasites, while still able to develop asexual stages, failed to produce oocysts, resulting in a 98% reduction of transmission through mosquitoes [90]. This observation suggests a potential therapeutic target that could interrupt transmission and argues for a complete in vivo characterization of K^+^ channels in *Plasmodium* that could offer new insights into the role of ion regulation in the parasite’s life cycle.

Calcium is the most universal second messenger, mediating signaling pathways that control cell cycle, gene expression, excitability, trafficking, secretion, and cell death [2,56,91–94]. Changes is free cytosolic Ca^2+^ also facilitate cell motility, differentiation, and tumorigenesis [95–97]. In intracellular parasites, host cell invasion [4,48,98] and egress are also Ca^2+^ dependent [54,99–103]. The evidence of Ca^2+^ uptake from the extracellular medium and the interaction of this ion influx with the intracellular stores is overwhelming, pointing out the presence of Ca^2+^-permeable channels at the plasma membrane [4,53,104–108]. L-type nifedipine-sensitive Ca^2+^ conductances have been described in *T*. *cruzi* [109–111], *Leishmania* [105,110,112], and *T*. *gondii* [4]. In agreement with this functional evidence, putative genes for L-type voltage-gated Ca^2+^ channels (VGCCs) have been identified in the genome of these parasites [113]. Rodriguez-Duran and colleagues recorded the activity of plasma membrane vesicles purified from *T*. *cruzi* epimastigotes and reconstituted into liposomes [110]. They established the presence of Ca^2+^ currents activated by sphingosine, miltefosine, and Bay K 8644, an activator of L-type VGCC. While all the sphingosine-stimulated activity was blocked by nifedipine, this compound only partially blocked currents elicited by miltefosine, suggesting either a difference in the activation mechanism or, more plausibly, the presence of more than one type of channel. While the permeability sequence agrees with previously characterized L-type VGCC, the channels in *T*. *cruzi* are not activated by voltage, indicating a difference in the gating mechanism. The only sequence identified in the *T*. *cruzi* genome as a putative VGCC is TcCLB.504105.130, with homologs in *Leishmania* and *T*. *brucei* (Table 1). Ca^2+^ uptake stimulated by sphingosine and miltefosine has also been described in *L*. *mexicana* and *L*. *donovani* [105,112]. In *T*. *brucei*, a putative VGCC called FS-179 has been localized to the flagellar membrane, but no functional studies to verify the biophysical properties of this protein have been conducted [59,114]. Definitive molecular evidence linking the nature of these currents with specific proteins is an important step to understand how trypanosomatids regulate Ca^2+^ flux at the plasma membrane.

*Plasmodium* Ca^2+^ currents are refractory to L-type channel blockers [107,115], and no putative VGCC sequences were found in its genome. Instead, possible homologs to transient receptor potential (TRP) channels are present in *P*. *falciparum*, *P*. *vivax*, and *P*. *knowlesi* [113], but their expression and function remain uncharacterized. In *T*. *gondii*, a recently described TRP channel, TgTPPL-2, mediates Ca^2+^ influx in tachyzoites [52]. This nonselective cation channel, localized in the plasma membrane and the endoplasmic reticulum (ER), contributes with approximately 50% of the uptake stimulated by physiological extracellular concentrations of Ca^2+^ and is not blocked by nifedipine, indicating that additional channels must be responsible for the residual Ca^2+^ influx [52]. Parasites in which TgTPPL-2 expression has been down-regulated showed reduced growth and impaired invasion and egress. When expressed in HEK-3KO cells, this channel also mediated currents at the ER, suggesting its possible role in the release of Ca^2+^ from intracellular stores [52]. This work provided the first molecular evidence linking a membrane channel with the observed Ca^2+^ influx in *T*. *gondii*, but it also underscores the need for electrophysiological recording methods that can be applied directly on the parasites, as the biophysical properties observed in heterologous expression system may not fully represent the activity of the native channels.

Chloride is the most abundant anion in nature, with extracellular concentrations of 120 mM and intracellular values ranging from 10 to 40 mM, depending on the cell type [6]. Plasma membrane chloride channels are responsible for the permeation of Cl^−^ and other anions, counterbalancing the net positive charges resulting from cation flows, and ensuring electroneutrality of cotransporters. As such, chloride channels are key regulators of cell volume, excitability, contractility, cell survival, and transepithelial transport [3,116,117]. Recently, the role of Cl^−^ as a regulator of cell signaling and cell proliferation has been associated with the progression of cancer and immune activation [3,118]. Chloride channels can be activated by voltage (CLC channels), volume (volume-regulated anion channels (VRACs)), ligands (GABA and glycine), or Ca^2+^ (calcium-activated chloride channels (CaCCs)). A particular type is the CFTR (cystic fibrosis transmembrane conductance regulator), epithelial chloride channel regulating secretion and water balance in multiple organs, and whose loss of function mutations underlie cystic fibrosis pathologies [119,120]. Localized either at the plasma membrane or in intracellular organelles, their function is required for adequate ion homeostasis.

In protozoans, there is limited information regarding expression and activity of plasma membrane Cl^−^ channels, with only one protein characterized in *Entamoeba* [121] (Table 2). EhCLC A is localized in the plasma membrane of trophozoites and shares homology with CLC-2 channels, including its permeability sequence and lack of modulation by pH, supporting its role as an electrogenic Cl^−^ channel and not as an antiporter. A second sequence named EhCLC B was also identified, but its characterization is still pending [121]. Patch clamp recordings of *Giardia intestinalis* trophozoites showed the activation of chloride currents compatible with CLC-2-type channels [122]. pH had no effect on the magnitude of the currents but did modify the voltage sensitivity. Unlike EhCLC A, only responsive to SITS (4-acetamido-4-isothiocyanatostilbene-2,2-disulfonic acid), the channels in *G*. *intestinalis* were blocked by canonical Cl^−^ channel blockers DIDS (4,4′-diisothiocyanatostilbene-2,2′-disulfonic acid) and niflumic acid but not by SITS. The putative genes encoding for these channels have not been identified, but *G*. *intestinalis* mRNA injection in *Xenopus laevis* oocytes produced at least three types of chloride currents with different profiles: a CLC-2-type, a Ca^2+^-activated, and a volume-responsive chloride channel. These three channels showed distinct voltage dependency, gating, and selectivity, providing solid functional evidence of their independent nature. The difference in their pharmacological profiles is a useful tool for functional analysis since the molecular identity and localization of these channels have not been established yet. Injection of mRNA from *Leishmania amazonensis* also elicited anionic currents sensitive to DIDS and niflumic acid, but the interpretation of this data is difficult due to the experimental conditions and the presence of endogenous Cl^-^ currents in the oocytes [123]. Certainly, several classes of Cl^−^ channels must be present in the plasma membrane of protozoan parasites. Their molecular identification and characterization would provide a missing piece in the proposed model of membrane exchange that supports ionic balance and secondary transporters required for survival, homeostasis, and infectivity [45,124].

### Aquaporins

Movement of water across membranes is a fundamental cellular process necessary for cell survival. Net flow of water results from changes in ion and osmolyte concentrations between the intracellular and the extracellular space, which causes an osmotic imbalance and drives fluid movement. Most of the water mobilization occurs through specialized water channels called aquaporins (AQP). Initially found in human erythrocytes and kidney cells [125], they have been described in most organisms, and their main role is to regulate cell volume [1,125,126]. Water channels can be classified in three groups: canonical aquaporins, only permeable to water; aquaglyceroporins that allow flow of glycerol, urea, and other small molecules; and superaquaporins, found intracellularly mostly in mammals [127].

**Fig 2:**
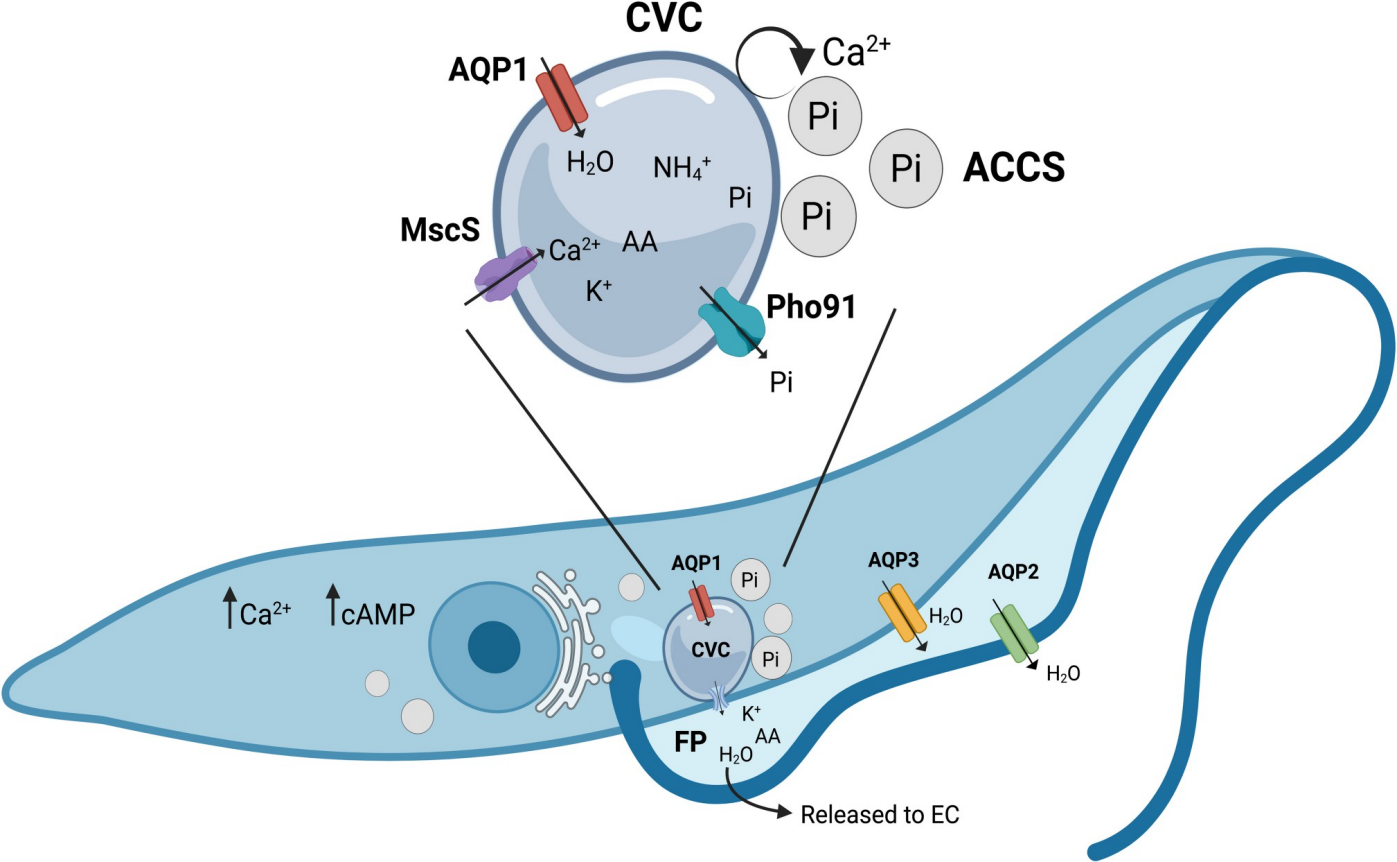
Mechanisms of osmoregulation in *Trypanosoma cruzi*. In *T*. *cruzi*, cell volume regulation is controlled by the CVC and the ACCS (reviewed in [247]). Both organelles are rich in ions accumulated by the activity of pumps located on their membranes. Upon hypoosmotic stress, ACCS fuse with the CVC transferring Pi, Ca^2+^, and AA. This concentration of osmolytes drives water into the vacuole via AQP1, causing swelling of the bladder that can be sensed by a mechanosensitive channel (MscS). Contact between the CVC membrane and the flagellar pocket induces the formation of a transient pore that mediates the discharge of water and osmolytes to the EC space. Some ions, like Pi, are not released but instead recovered by transporters (Pho1). These events are accompanied by increase in cAMP and a rise in cytosolic Ca^2+^ required for volume recovery. *Trypanosoma brucei* does not possess a contractile vacuole and regulates cellular volume via aquaporins, with AQP2 residing in the flagellar membrane and AQP3 at the plasma membrane. *Figure created with BioRender.com*. AA, amino acid; ACCS, acidocalcisome; CVC, contractile vacuole complex; EC, extracellular; Pi, phosphate.

In protozoan parasites, the number, expression, and localization of AQP varies from only one in *T*. *gondii* [128] and *P*. *falciparum* [129], to five putative AQPs in *Leishmania* [130–133]. It has been proposed that this variation correlates with the environmental challenges faced during the life cycle and the capacity of the parasites to respond to them [134]. In *T*. *brucei*, TbAQP1 and TbAQP2 localize to the flagellar membrane and flagellar pocket, respectively (Table 1), while TbAQP3 is restricted to the plasma membrane. In *L*. *major*, LmAQP1 is also found in the flagellum, suggesting a role of this structure in sensing osmotic conditions. In *T*. *cruzi*, the only characterized AQP, TcAQP1, mediates water flow to the contractile vacuole complex (CVC), an organelle that regulates cell volume in the parasites (Fig 2) [135,136]. Functional studies showed that most AQPs found in protozoan parasites, except for TcAQP1 [135], are permeable to water as well as glycerol and other small solutes including urea, ammonia, and arsenite. Glycerol permeation through AQPs could play a role in regulating glycosomal activity and energy metabolism [137,138].

AQPs are considered major intrinsic proteins (MIPs), membrane channels that mediate the exchange of water and small solutes. As such, they also play a role in permeability to antiparasitic compounds and have been postulated as potential drug targets [130,139]. In *Leishmania* [132] and *T*. *brucei* [140], AQPs mediate the uptake of trivalent antimonial compounds and down-regulation of LmAQP1 correlates with resistance to antileishmanial drugs such as Pentostam [132,141], while TbAQP2 expression is linked to melarsoprol-pentamidine cross-resistance [142].

Evidently, the physiological functions of AQPs in protozoans go well beyond cell volume regulation, possibly also regulating metabolisms and drug susceptibility as well. Some protozoans can manipulate the expression of AQPs in the host cells. Hepatocytes infected with *P*. *vivax* showed increased AQP3 expression, and active recruitment of the host’s AQP3 to the parasitophorous vacuole membrane (PVM) during liver and blood stage development [143,144]. Down-regulation of AQP3 by RNAi or inhibition by auphen drastically reduced the parasite load in liver cells. Analysis of *P*. *falciparum-*infected RBCs showed similar recruitment of AQP3 to the PVM, and treatment with auphen reduced parasitemia by 75% [144]. While the mechanism of translocation from the plasma membrane to the PVM has not been established, the temporal profile of recruitment early during schizont and erythrocytic stages suggest a role of the channel as a nutrient uptake pathway [143]. Recently, transcriptomic analysis of permissive and nonpermissive hepatocytes has identified AQP9 as a host cell factor required for sporozoite invasion [145]. Silencing or chemical inhibition of host APQ9 drastically reduced hepatocyte invasion by *P*. *falciparum* and *P*. *berghei* without compromising further intracellular development of liver stages. The role of AQP9 during invasion is unclear, but it is possible that osmotic changes at the plasma membrane, elicited by water influx, could indirectly modify actin polymerization observed during invasion [145] an interesting hypothesis that requires further exploration.

### Mitochondrial channels

In mammalian cells, mitochondrial morphology, distribution, and functions have been extensively studied [146,147]. Originated by an endosymbiotic event [148], mitochondria possess a double-membrane structure that surrounds the matrix, where most of the proteins and the mitochondrial DNA (mtDNA) reside. The outer mitochondrial membrane (OMM) separates the cytosol from the intermembrane space, regulating interactions with other organelles and the exchange of metabolites and proteins [149]. The inner mitochondrial membrane (IMM) harbors metabolic pathways involved in oxidative phosphorylation, fatty acid oxidation, and small molecule transport [146]. Invaginations of the IMM give origin to the cristae, structures that fold toward the matrix and whose morphology and abundance depend on the presence of multimeric complexes including the ATP synthase and other electron transport chain (ETC) components, the mitochondrial contact site and cristae organizing system (MICOS) [150,151], and the protein importing systems [152].

Often referred to as the powerhouse of the cells for their role in ATP synthesis, mitochondrial functions expand beyond that of oxidation of fatty acids, redox balance, regulation of cell death, and calcium signaling modulation. The number, shape, and localization of these organelles, collectively known as mitochondrial dynamics [146,147], respond to cellular signals such as changes in metabolic demands, immune challenges, and the presence of intracellular pathogens, among others [153–155].

In protozoans, mitochondrial morphology, function, and dynamics are dependent on the developmental stages of the parasites. Trypanosomatids possess a single mitochondrion, with its mtDNA organized in maxi and mini circles forming the kinetoplast. The OMM and IMM extend throughout the body of the parasites, and the abundance of cristae varies depending on life stages. *T*. *brucei* bloodstream trypomastigotes are mostly glycolytic, and their mitochondria, although essential [156,157], are not as developed as in the procyclic forms that rely heavily on oxidative metabolism [158]. *T*. *gondii* tachyzoites [159,160] and *Plasmodium* blood and liver stages also have a single mitochondrion that undergoes extensive branching and remodeling during cell division [161,162]. In contrast, *Cryptosporidium*, *Entamoeba*, and *Giardia* possess mitochondria that have lost most of their genome and are unable to perform oxidative phosphorylation due to an incomplete ETC [163–167]. These organelles, called mitosomes, are required for parasite survival and play essential roles in energy production by substrate-level phosphorylation, lipid metabolism, and assembly of Fe-S clusters. Similarly, in *T*. *vaginalis* and other trichomonads, organelles known as hydrogenosomes [168] evolved from a mitochondrial-like ancestor but completely lost their genome and most ETC-related proteins [167,169]. Instead, they produce ATP by substrate-level phosphorylation and protons by the activity of an Fe hydrogenase [170]. Hydrogenosomes possess a robust protein import machinery with similar components to those found in mammalian mitochondria [171–173].

Despite the vast diversity of forms and functions, mitochondria and related organelles in protozoans possess a membrane potential that must be maintained for the parasites to survive. In *T*. *brucei*, the mitochondrial membrane potential (_mt_Vm) has been estimated to be approximately −130 mV both in procyclic and bloodstream forms [174]. Similarly, *T*. *cruzi* epimastigotes have a _mt_Vm of about −140 mV, while *Leishmania* promastigotes showed values close to −120 mV [175,176]. In *Plasmodium* trophozoites [177,178] and *T*. *gondii* tachyzoites [179,180], reported _mt_Vm values are similar to those in trypanosomatids. The negative _mt_Vm depends on two factors: the maintenance of the proton gradient between the intermembrane space and the mitochondrial matrix generated by the activity of the ETC and the ATP synthase, and the strict control of the permeability of the IMM [181]. In contrast, the OMM is highly permeable to small molecules and ions, mostly due to the presence of porin-like proteins such as the translocase of the outer membrane complex (TOM) [182,183] and the voltage-dependent anion channel (VDAC) [184]. First described in 1976 using planar bilayers with mitochondrial membranes isolated from *Paramecium* [184], VDAC, it is the most abundant protein in the OMM [185]. Under physiological conditions VDAC is a nonselective porin, preferentially permeable to chloride but also able to conduct ATP, K^+^, and Ca^2+^ depending on the transmembrane voltage [186]. The channel allows for the passage of noncharged solutes from 3 to 6 kDa in size [187], constituting an important regulator of metabolic flux between the cytosol and the mitochondria [188]. The role of VDAC has been extensively studied in eukaryotes and includes the regulation of mitochondrial metabolism, apoptosis, protein import, and modulation of mitochondrial morphology [189–191], but the extent to which these discoveries apply to parasitic protozoa is unclear. Importantly, VDAC plays a fundamental role in the interaction of mitochondria with other organelles. VDAC1 and VDAC2 interact with IP_3_ receptors localized at the mitochondria-ER contact sites (MERCS) and is required for Ca^2+^ flux [192,193], while VDAC2 and ryanodine receptor (RyR) 2 regulate Ca^2+^ dynamic in cardiac cells [194]. Recently, Mallo and colleagues showed that in *T*. *gondii*, TgVDAC localizes to MERCS, and depletion of the channel reduces the number of contact sites, leading to ER fragmentation [195]. Down-regulation of TgVDAC in tachyzoites caused replicative defects accompanied by aberrant mitochondrial morphology. Surprisingly, while TgVDAC depletion disrupted protein import to the mitochondria, no defects in Ca^2+^ homeostasis were observed and only a marginal decrease in ATP/ADP ratio was reported [195]. These results support a partially conserved role of TgVDAC in *T*. *gondii*, mainly regulating mitochondrial dynamics and inter-organelle contact.

In *T*. *brucei*, two putative VDAC sequences have been identified (Table 1) [196]. The porin was found as oligomers of an apparent molecular weight of 212 kDa, corresponding to a hexameric or heptameric complex as previously reported in other cells [189]. Knockout of VDAC1 in procyclic forms leads to growth defects only under conditions that favor oxidative phosphorylation, showing no difference in fitness if glucose is present. This study suggests that VDAC is not required for survival in parasite stages that rely in glycolytic production of ATP [197]. In agreement with metabolic differences previously reported [198,199], Singha and colleagues showed a 5-fold higher expression of VDAC in procyclics compared to bloodstream forms [200], but in this work, authors reported a decrease in growth and mitochondrial ATP production in both life stages upon down-regulation of VDAC [200]. Despite these discrepancies, it is clear that VDAC plays a role in metabolite import to the mitochondria in *T*. *brucei*. Putative VDAC sequences are present in the genome of *T*. *cruzi*, *Leishmania*, and *Plasmodium* awaiting for experimental interrogation of their roles in the biology of these parasites.

The proton motive force across the IMM responsible for the membrane potential also creates a driving force for Ca^2+^ uptake, making mitochondria one of the most important Ca^2+^ stores in the cell. Through fine-tuning of signaling and Ca^2+^ modulation, the mitochondria control cell fate, triggering either necrosis or apoptosis in response to level and duration of cytosolic Ca^2+^ increases. The accumulation of Ca^2+^ in the mitochondria requires the permeation through the OMM, possibly mediated by VDAC [192–194], and the IMM, via the mitochondrial calcium uniporter (MCU) [201]. The MCU is a hetero-oligomeric protein complex whose constituents reside embedded in the IMM and protrude into the intermembrane space. The MCU is the pore-forming protein that mediates Ca^2+^ import. It associates with regulatory proteins MICU1 and MICU2, as well as EMRE (essential MCU regulator), an essential subunit that facilitates the interaction of MCU and MICUs (reviewed in [202]). An alternative pore-forming subunit, MCUb, can associate with MCU, reducing its transport capacity, as MCUb is not able to permeate Ca^2+^ in mammalian cells. In trypanosomatids, mitochondrial Ca^2+^ uptake mediated by an MCU-like mechanism was postulated in 1989 in *T*. *cruzi* epimastigotes [203,204] and later also found in *Leishmania* [175] and *T*. *brucei* [174]. Molecular identification of the proteins responsible for this Ca^2+^ influx will follow 30 years later, with the characterization of the MCU complex in *T*. *brucei* [205,206] and *T*. *cruzi* [207–209]. In these parasites, the core subunits MCU, MCUb, and MICU1/2 have been localized to the mitochondria, where they were shown to be essential for Ca^2+^ uptake and bioenergetic functions. No homologs of EMRE have been found in trypanosomes. Surprisingly, while mammalian MCUb is a negative regulator of Ca^2+^ uptake, *T*. *cruzi* homolog TcMCUb facilitates mitochondrial Ca^2+^ uptake, and its ablation negatively impacts parasite survival, differentiation, and infectivity [207]. *T*. *brucei* homolog (TbMCUb) has similar functions, and its down-regulation impairs mitochondrial Ca^2+^ uptake without causing changes in _mt_Vm or cell growth [206]. Two additional subunits TbMCUc and TbMCUd, uniquely identified in *T*. *brucei*, are expressed and localized to the mitochondria, directly interacting with TbMCUb and TbMCU [206]. RNAi of any of the TbMCU subunits caused a decrease in Ca^2+^ uptake without further detriment to the cells, unless they were deprived of glucose. While this may suggest a certain degree of redundancy on the formation of the MCU complexes in *T*. *brucei*, the inability to complement the function with alternative subunits indicates unique roles for each of the components. Supporting this idea, the elimination of TcMCU or TcMCUb in *T*. *cruzi* impacted the ability of the mitochondria to capture Ca^2+^ without affecting the membrane potential, but only TcMCUb knockouts showed reduced growth, respiration rates, differentiation, and infectivity [207]. A similar phenotype was observed when MICU1 or MICU2 were eliminated, but their role in the regulation of the MCU differs from reports in mammalian cells, as *T*. *cruzi* MICUs seem to work as stabilizers of the complex rather than as gatekeepers [210].

The activation of the MCU causes a transient Ca^2+^ increase in the mitochondria in the order of 50 to 100 mM, while the average cytosolic concentration is around 100 nM. This apparent discrepancy, together with the reported low affinity of the MCU [201], posed the question of how was the Ca^2+^ uptake possible. Multiple evidences have shown that the influx occurs at cellular microdomains where the mitochondria can interact with the ER [211] or with the plasma membrane [212] and that the activation of IP_3_R, RyR or other channels provides a local Ca^2+^ increase that can be captured by the MCU complex [192,193,213]. In trypanosomatids, IP_3_Rs have been found in the acidocalcisomes instead of the ER [214,215], suggesting a possible interaction between these Ca^2+^-rich organelles and the mitochondria [216,217].

The trypanosome MCU complex fulfills crucial roles in Ca^2+^ uptake and metabolism, as it has been shown in mammalian cells, but it also seems to affect replication, differentiation, and infectivity of the parasites. The presence of new MCU subunits, found exclusively in trypanosomes, may offer a window for selective targeting of the parasites, as Ca^2+^ homeostasis has been postulated as a potential area for drug development [218].

In apicomplexans, motility, invasion, and egress are strongly dependent on Ca^2+^ [4,55,106,219]. Despite some reports of sensitivity to the MCU blocker ruthenium red [220], there is no genomic evidence of an MCU complex in either *Toxoplasma* or *Plasmodium*, suggesting the loss of these proteins, as it has been also observed in mitosome-harboring protozoans and some yeast [221]. Whether mitochondrial Ca^2+^ plays an important role in regulating metabolic and cellular functions and how is this ion pool managed in apicomplexans are still pending questions.

### Channels in other organelles

Intracellular compartments maintain their composition by harboring a differential set of proteins that support specialized functions. In protozoans, ER, acidocalcisomes, and mitochondria are the main intracellular Ca^2+^ stores, controlling the amount of free cytosolic Ca^2+^ and cellular functions linked to Ca^2+^-dependent signaling [216]. In apicomplexans, two additional organelles participate in Ca^2+^ homeostasis: the food vacuole of *Plasmodium* [222] and the plant-like vacuole in *T*. *gondii* [223], while in *T*. *cruzi*, the CVC has been postulated as a Ca^2+^ store [224]. Acidocalcisomes are acidic organelles rich in phosphate found in trypanosomatids [98,225,226], apicomplexans [227–229], and other microorganisms [230] including bacteria [231], nonparasitic protozoa [232], and algae [233]. In *T*. *brucei* and *T*. *cruzi*, they accumulate calcium through a Ca^2+^-ATPase abundantly expressed in their membrane [234–236], while efflux is mediated by IP_3_ receptors [214,215]. Nuclear patch clamp recordings of DT40–3KO cells expressing TbIP_3_R (*T*. *brucei* IP_3_ receptor) showed that the channel is modulated by phosphate, with an increase in the current and open probability in the presence of orthophosphate or pyrophosphate, while polyP3 and acidic pH had an inhibitory effect [237]. These results suggest that TbIP_3_R could be activated under hypoosmotic stress conditions, when the acidocalcisomes show alkalinization and polyP hydrolysis [238,239]. Importantly, down-regulation of TbIP_3_R reduces cell growth in procyclic and bloodstream forms, also decreasing infectivity in vivo [215]. Similarly, knockout of the IP_3_R in *T*. *cruzi* causes severe reduction of epimastigotes growth and intracellular amastigote replication, decreasing metacyclogenesis and infectivity. The elimination of TcIP3R has a profound effect in mitochondrial functions with decreased Ca^2+^ uptake, oxygen consumption and citrate synthase activity, increased AMP/ATP ratio, and induction of autophagy. This phenotype is remarkably similar to the one observed in parasites where the MCU subunits were ablated [207,209], strongly supporting the hypothesis of acidocalcisome-mitochondrion contacts regulating Ca^2+^ dynamics [240] and providing additional evidence of the role of Ca^2+^ channels as regulators of parasite fitness in trypanosomes [216].

In apicomplexans, despite the responses elicited by IP_3_ [49,241] and cyclic ADP ribose [242], and the presence of PLC signaling pathways [243,244], no molecular evidence of IP_3_R or RyR expression has been found, suggesting that the release of Ca^2+^ from intracellular stores could be mediated by a highly divergent type of IP_3_R or by a different type of IP_3_-responsive channel [245], exciting hypotheses that require further experimental support.

Acidocalcisomes accumulate large amounts of phosphate as polyP polymers. These anionic molecules interact with inorganic and organic cations, translocated to the lumen by cation transporters and exchangers (reviewed in [240]). An inwardly rectifying K^+^ channel (TbIRK; Table 1) has been localized to the acidocalcisomes of *T*. *brucei*, contributing to the cation influx [246]. As expected by the lack of a conserved selectivity filter, this channel has a relative permeability P_K_/P_Na_ around 7, quite small considering that selective K^+^ channels have a preference for K^+^ over Na^+^ of hundred to thousand folds [15]. Its down-regulation had no effect on the growth of procyclics but could not be evaluated in bloodstream forms due to low RNAi efficiency, leaving an open question about the physiological role of this channel.

In *T*. *cruzi*, acidocalcisomes have been shown to fuse with the CVC, transferring ions and proteins to this compartment [136]. The accumulation of ions and organic osmolytes in the CVC attracts water, causing periodic discharge of its content to the extracellular space and regulating the parasite’s volume (Fig 2). The CVC operates in cycles of systole and diastole, which, under isotonic conditions, cause a discharge event approximately every 90 seconds. The frequency of CVC emptying depends on the osmotic conditions, increasing under hypoosmotic stress [247]. It has been postulated that sensing of the filling state must be mediated by mechanosensitive elements. Recently, a K^+^- and Ca^2+^-permeable mechanosensitive channel residing in the CVC (TcMscS) has been identified in *T*. *cruzi*. The channel participates in osmotic stress responses, Ca^2+^ homeostasis and regulation of cell differentiation and replication [111]. A homolog in *T*. *brucei* (TbrMscS) shows similar activity but is localized to the mitochondria. Putative mechanosensitive channels have been found in the genome of Apicomplexans, Trichomonads, and Amebae [248], but no functional studies have been conducted so far. Recently, a two-pore channel (TgTPC) has been identified in the *T*. *gondii* [57]. TPCs are cation channels abundantly expressed in the endolysosomal system [249] where, due to their mechanosensitivity, they participate in organelle biogenesis, osmotic responses and volume regulation [250,251]. In *T*. *gondii*, TgTPC is exclusively found in the apicoplast, an endosymbiotic organelle that harbors multiple metabolic pathways required for parasite division [252–254]. Li and colleagues showed that TgTPC is needed for proper formation of apicoplast-ER contact sites and seems to mediate the transference of Ca^2+^ between these organelles [57]. Parasites lacking TgTPC lose their apicoplast and have a severe growth defect that resembles the phenotype of other apicoplast-defective mutants [255], suggesting that TgTPC is necessary to maintain the integrity of the organelle and the fitness of the parasite.

The participation of intracellular channels in organellar crosstalk is emerging as a new physiological role of these proteins in protozoan parasites. The localization, properties, and regulation of these channels is proving to be as unique as the biology of the parasites themselves, and further studies are required to explore the potential of these proteins as selective drug targets.

## Master manipulators: Ion channels at the interface with the host cell

A hallmark of parasitic lifestyle is the ability to manipulate the host for the benefit of the parasite and protozoans have developed multiple strategies to ensure their supply of “goodies.” The interaction between parasites and their mammalian hosts causes extensive changes to the host cells, mediated by secretion of extracellular vesicles, immunomodulatory effects, changes in gene expression, and modification of cell permeability. The most extensively studied example is the remodeling of erythrocytes during *Plasmodium* infection, which involves the formation of new permeation pathways (NPPs) [256]. Upon RBC invasion, the parasites establish a parasitophorous vacuole (PV) where they reside for their intraerythrocytic development. Early on during PV formation, parasite-secreted proteins form a pore known as *Plasmodium* translocon of exported proteins (PTEX) [257–259]. This complex facilitates the export of proteins that completely change the physiology of the RBC. This “exportome,” consisting of more than 300 proteins and can be divided into four major groups: heat-shock proteins, proteins involved in lipid homeostasis, essential kinases, and proteins that mediate nutrient acquisition (reviewed in [260]). The result is a modification of the RBC intracellular concentrations of Na^+^ and K^+^ [261] with increased permeability to organic cations [262], organic osmolytes [263], and anions [264]. Most of these NPPs are due to the plasmodial surface anion channel (PSAC), a multimeric complex produced by the parasites and exported to the RBC membrane [265]. PSAC contains the pore-forming subunit CLAG3 and two associated proteins RhopH2 and RhopH3 [266], required for trafficking to the cell surface [267,268]. Two main purposes have been postulated for PSAC; one is nutrient uptake of essential molecules required for intracellular growth [264,265,269,270], but the marginal fitness defect caused by CLAG3 knockout in vitro suggests additional roles [271]. Upon insertion of PSAC in the RBC membrane, intracellular Na^+^ concentration increases 10 times, while K^+^ decreases significantly. It is possible that the ionic environment modification is what fuels nutrient uptake and membrane potential regulation by sodium-dependent secondary transporters, located at the parasite cell membrane [62,272,273]. Electrophysiological analysis of PSAC revealed a voltage sensitive anionic channel with unusually small conductance and with very low permeability to Na^+^ [264,274]. Paradoxically, it has a broad selectivity for sugars, amino acids, purines, and vitamins [269,275–277]. The identity of the pore-forming proteins involved in the PSAC activity is still matter of debate [24,25,278]. Multiple studies addressing the composition and characteristics of the NPPs have given origin to two alternative models. The first one postulates CLAG3 as the pore-forming unit responsible for PSAC conductance [264,265,267,270], while the second favors the idea that CLAG3 interacts with chloride channels from the host cell, modulating the activity of the NPPs [279–282].

Whether PSAC functions to mediate uptake directly or indirectly, what is certain is that, once in the RBC cytosol, nutrients must cross the PV to reach the parasites. In erythrocytic and liver stages, only one protein has been identified as a nutrient pore located in the PV, the exported protein 2 or EXP2. EXP2 is a size-selective channel that allows small molecules up to 1.4 kDa to traverse the PVM, constituting the main permeation pathway for amino acids and sugars [283,284]. It has a large conductance of around 300 pS, and several subconductance states [283]. A particular aspect of EXP2’s function is its ability to import nutrients when it forms homomeric complexes, or to interact with Hsp101 and PTEX150 to assemble the PTEX protein-exporting pore [283,285]. While EXP2 seems to be sufficient to form a conductive pore, optimal activity at the PVM requires exported protein 1 (EXP1) [286]. Association between EXP1 and EXP2 is necessary for nutrient uptake, but not for the exporting functions of EXP2. Homologues of EXP2 have been found in *T*. *gondii*, where GRA17 and GRA23, dense granule proteins secreted by intracellular tachyzoites, form a nutrient-permeable pore with similar characteristics to EXP2 [287]. The elimination of EXP2 or GRA17 resulted in severe reduction of parasite growth, indicating their essentiality for intracellular proliferation [283,287–289]. Thus, PV forming apicomplexans seem to have developed a common strategy for scavenging resources from their host cell, by establishing large nonselective pores in the membrane of the PV.

In trypanosomatids with intracellular developmental stages, the PV provides a protective environment for *Leishmania* parasites replicating inside macrophages [290,291], while in *T*. *cruzi*, the PV is a transient structure [292]. *Leishmania* promastigotes infect phagocytic cells by promoting their own endocytosis. After incorporation into the phagolysosome, promastigotes differentiate to amastigotes that replicate intracellularly and live in their protective PV [293]. The maturation of the PV involves an active exchange with the endolysosomal system (reviewed in [39]). Fusion of vesicles with the PV provide an environment rich in sugars, amino acids, and lipids required for amastigote replication [294–297]. Thus, active nutrient scavenging is restricted to arginine and iron [298], which seem to be accumulated in the PV by the mammalian arginine transporter SLC38A9 and Nramp1, a putative iron pump [298]. The evidence suggest that *Leishmania* has established a different strategy for nutrient acquisition, promoting the insertion of host cells transport proteins into the PVM, instead of producing and secreting its own pore-forming proteins, as it is the case in apicomplexans.

*T*. *cruzi* trypomastigotes invade cells either by a lysosomal membrane repair-induced mechanism [299] or by interaction with surface molecules and activation of PIP_3_-dependent signaling pathways [300]. In both cases, the parasite establishes a PV within the first two hours postinvasion and differentiates into intracellular amastigotes. Shortly after, there is a marked increase in the pH of the acidic vacuole followed by fragmentation of the PV and escape of the amastigotes to the cytosol, where they replicate with full access to host cell nutrients [301]. The disassembly of the PV is presumably triggered by the secretion TcTOX, a protein that has pore-forming activity at acidic pH but whose molecular identity has not been elucidated yet [301].

Each of the strategies summarized above represent a unique form of parasite interaction with their host cells that has evolved to provide a suitable environment for intracellular development. The disruption of these mechanisms for nutrient acquisition affects the growth of the parasites, deserving further attention as they could also be an obvious molecular target for the development of new therapeutic alternatives.

## Future perspectives: Ion channels as potential drug targets

Infections caused by protozoan parasites are still a major public health problem, mostly in developing countries where coinfections with other microorganisms are common. Considering the high prevalence of *T*. *gondii* (around 30%) and *Plasmodium* infections averaging over 200 million a year, the combined burden of diseases caused by protozoans affects almost half of the world population. The therapeutic options for treating infections caused by these parasites are limited and have complex administration regimes that rely on drug combinations and long treatments. Finding affordable, selective, and effective oral compounds that do not elicit resistance is the goal of current drug development efforts.

**Fig 3:**
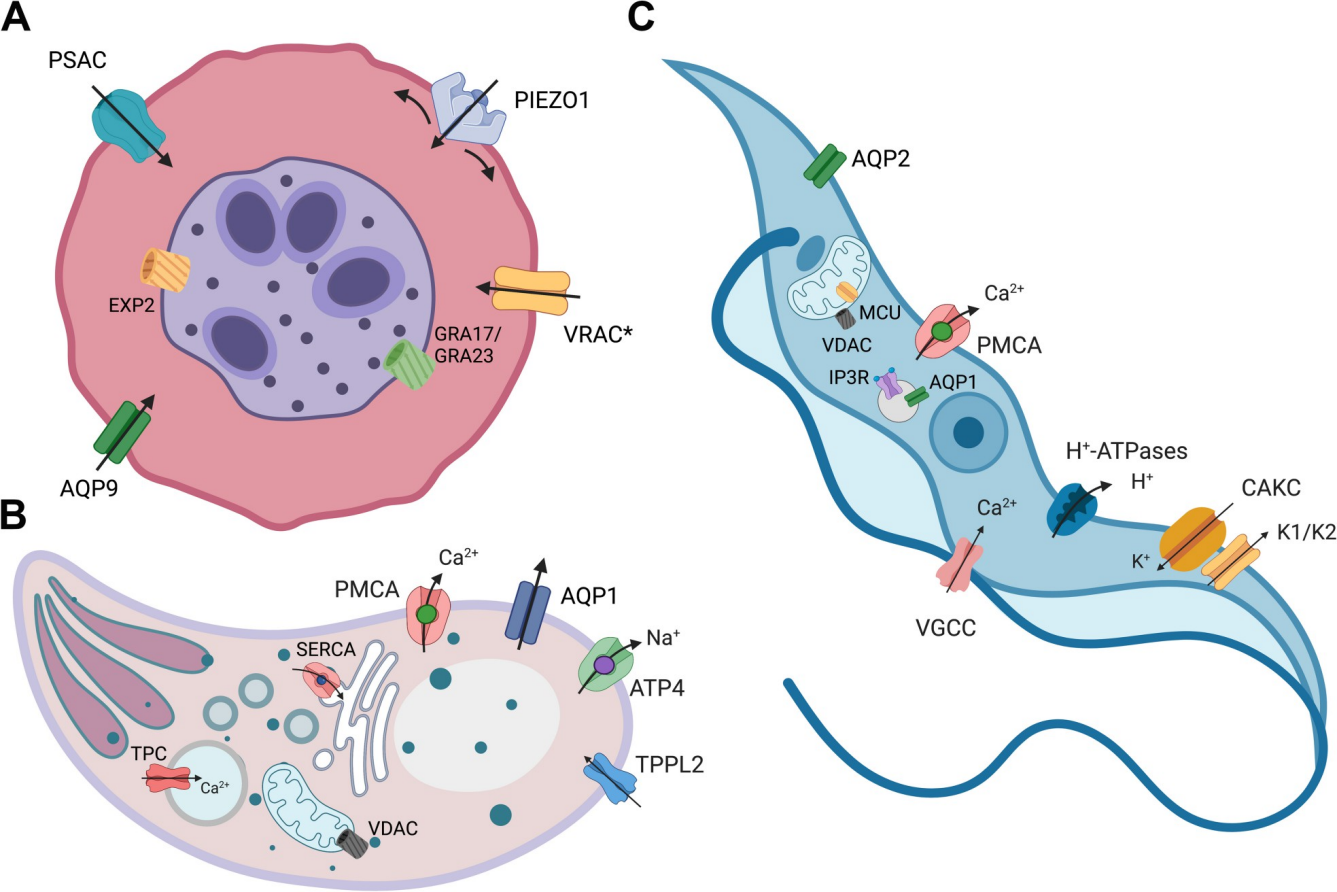
Ion channels postulated as targets for antiparasitic drug development. Three sets of ion channels have been suggested as potential drug targets against protozoan parasites: (1) proteins located in the host cell membrane and the PV **(A)**; (2) those expressed at the plasma membrane of the parasites; and (3) channels found in intracellular organelles. (**A)** *Plasmodium* parasites residing intracellularly export proteins like PSAC to the host cell. Thus, parasites control the import of nutrients while also exploiting proteins from the host cell to create favorable conditions for their growth. AQP9 and PIEZO1 activity increases in infected RBC, and VRAC activity is stimulated in liver cells by *Plasmodium* infection. Blockage of these proteins severely impacts parasite growth, suggesting their potential as drug targets. Similarly, interference with the activity of pore-forming proteins in the PVM such as EXP2 in *Plasmodium* and GRA17/GRA23 in *Toxoplasma gondii* blocks nutrient import and parasite growth. (**B) In apicomplexans,** blockers of the Na^+^ pump ATP4 impact parasite growth and PfATP4 has been shown susceptibility to a large number of compounds. Plasma membrane calcium pump (PMCA) and TPPL2 Ca^2+^ channels are essential for *T*. *gondii* tachyzoite growth, and so it is the SERCA pump in the ER, showing that Ca^2+^ homeostasis is a promising target for drug development. Additional proteins worth to explore in *T*. *gondii* are the apicoplast two-pore channel TPC and mitochondrial porin VDAC. (**C) In trypanosomatids**, Ca^2+^ controlling proteins including calcium channels (VGCC, IP_3_ receptors, VDAC, and MCU) and the PMCA have been proposed as druggable targets. Expression of H^+^-ATPases and K^+^ channels with very low homology with mammalian proteins could offer an opportunity for the development of selective inhibitors. Modulation of AQP activity is associated with resistance to anti-leishmania therapy and could be exploited as a permeation pathway for chemotherapy. *Figure created with BioRender.com*. ER, endoplasmic reticulum; MCU, mitochondrial calcium uniporter; PSAC, plasmodial surface anion channel; PV, parasitophorous vacuole; PVM, parasitophorous vacuole membrane; RBC, red blood cell; VDAC, voltage-dependent anion channel; VGCC, voltage-gated Ca^2+^ channel; VRAC, volume-regulated anion channel.

Analysis of therapeutic drugs currently in clinical use show that ion channels represent the second most frequent target after GPCR [302] and have been postulated as suitable drug targets against bacterial, viral, and parasitic infections [303–306]. L-type Ca^2+^ channel blockers verapamil, amlodipine, diltiazem, and nifedipine are the most prescribed treatment for cardiovascular diseases such as hypertension and angina [307,308]. Ion channel modulators are widely used to treat neurological and psychiatric disorders such as epilepsy, neuropathic pain, anxiety, and depression [309–314]. They have proven efficacious against helminths, with praziquantel activating TRP channels in *Schistosoma* [315], ivermectin targeting glutamate-activated chloride channels in a variety of nematodes [316,317], and imidazothiazoles and tetrahydropyrimidines acting as cholinomimetics [318,319]. While currently used antiprotozoal drugs do not target ion channels directly, artemisinin seems to inhibit PfATP6, the *P*. *falciparum* homolog of the SERCA pump [320], although some differences between in vitro and in vivo studies question this putative mechanism of action [321,322]. Atovaquone, another antimalarial often used in combination with proguanil, targets the ETC and collapses the mitochondrial membrane potential [323,324]. In trypanosomatids, Ca^2+^ channel blockers nifedipine, amlodipine, and amiodarone have shown antiparasitic activity [105,110,112], and Ca^2+^ homeostasis has been proposed as a cellular target for drug development [55,325,326]. Over the years, a number of channels and transporters essential for parasite survival have been identified (Fig 3). The disruption of mechanisms that facilitate nutrient and ion exchange in protozoans is a promising area for therapeutic advances. Targeting molecules localized at the plasma membrane of the host cells, the PV, or the parasites’ membranes can interrupt essential processes that sustain viability, growth, and infectivity. The obligated question is, Why aren’t we there yet? The study of ion channels and transporters in protozoans has faced important technical limitations derived from the difficulty of applying electrophysiological methods of patch clamp recording to small, often motile, cells. Additionally, while these powerful approaches have single molecule resolution, they are time consuming and require significant expertise [18], so they have been sidelined by high-throughput phenotypic screenings fueled by newly developed whole-genome CRISPR-based techniques. Undoubtedly, these studies have provided valuable information regarding essential genes and pathways in the parasites, but discrepancies between large-scale screenings and targeted functional studies argue for an integrative approach to correlate genetic and physiological data [24]. To speed up the evaluation of ion channels as suitable drug targets in protozoans, systematic studies addressing their structural and functional properties are paramount. Structural similarity rather than homology-based screenings, combined with high-throughput electrophysiology in heterologous and native membranes, would increase our capacity for the discovery of new ion channels and transporters that have evaded identification in protozoans [327,328]. These methods, combined with high resolution–low output tools such as cryo-electron microscopy and single-channel electrophysiology, would allow us to evaluate channels in situ, in a physiological context, and to pinpoint key differences that can be exploited to increase the selectively and potency of new compounds.

Finally, we must increase our commitment to translational applications of basic biology findings and incorporate new technologies to combat old foes. Repurposing of approved drugs, together with evaluation of new natural and synthetic compounds, offers a financially viable avenue for novel treatments against neglected parasitic diseases. The rapid growth of biologics as therapeutics, including antibodies to treat inflammatory, tumoral, and viral diseases (see complete list here: www.antibodysociety.org/antibody-therapeutics-product-data), brings a new tool that should be also considered as an alternative against parasitic diseases. For example, nanobodies targeting P2X channels have shown promising results against some types of cancer and inflammatory diseases [329–332].

Infectious diseases are still the most prevalent diseases in the world, and their treatment presents unique challenges driven by our limited understanding of host–pathogens dynamics, the influence of the host microbiomes, and the emergence of resistance, among other biological and socioeconomic factors. What we learn about the role of ion channels in protozoans and their potential as therapeutic targets can offer insights into new treatments for bacterial and fungal infections.

## References

[1] Bortner CD, Cidlowski JA. Ions, the Movement of Water and the Apoptotic Volume Decrease. Front Cell Dev Biol. 2020;8:611211. Epub 20201125. doi: 10.3389/fcell.2020.611211 ; PubMed Central PMCID: PMC7723978. 33324655 PMC7723978

[2] Lang F, Foller M, Lang K, Lang P, Ritter M, Vereninov A, et al. Cell volume regulatory ion channels in cell proliferation and cell death. Methods Enzymol. 2007;428:209–25. Epub 2007/09/19. doi: 10.1016/S0076-6879(07)28011-5 . 17875419

[3] Como M, Koppala BR, Hasan MN, Han VL, Arora I, Sun D. Cell Volume Regulation in Immune Cell Function. Activation and Survival Cell Physiol Biochem. 2021;55(S1):71–88. doi: 10.33594/000000331 . 33611867

[4] Pace DA, McKnight CA, Liu J, Jimenez V, Moreno SN. Calcium entry in Toxoplasma gondii and its enhancing effect of invasion-linked traits. J Biol Chem. 2014;289(28):19637–47. Epub 20140527. doi: 10.1074/jbc.M114.565390 ; PubMed Central PMCID: PMC4094074. 24867952 PMC4094074

[5] Plattner H, Verkhratsky A. Ca2+ signalling early in evolution—all but primitive. J Cell Sci. 2013;126(Pt 10):2141–50. Epub 20130531. doi: 10.1242/jcs.127449 . 23729741

[6] Valdivieso Á G, Santa-Coloma TA. The chloride anion as a signalling effector. Biol Rev Camb Philos Soc. 2019;94(5):1839–56. Epub 20190623. doi: 10.1111/brv.12536 . 31231963

[7] Petkov GV. Chapter 16—Ion Channels. In: Hacker M, Messer W, Bachmann K, editors. Pharmacology. San Diego: Academic Press; 2009. p. 387–427.

[8] Jan LY, Jan YN. Tracing the roots of ion channels. Cell. 1992;69(5):715–718. doi: 10.1016/0092-8674(92)90280-p . 1375539

[9] Hille B. Ionic channels in nerve membranes, 50 years on. Prog Biophys Mol Biol. 2022;169–170:12–20. Epub 20211129. doi: 10.1016/j.pbiomolbio.2021.11.003 ; PubMed Central PMCID: PMC8977236. 34856230 PMC8977236

[10] Global, regional, and national disability-adjusted life-years (DALYs) for 359 diseases and injuries and healthy life expectancy (HALE) for 195 countries and territories, 1990–2017: a systematic analysis for the Global Burden of Disease Study 2017. Lancet. 2018;392(10159):1859–1922. doi: 10.1016/S0140-6736(18)32335-3 ; PubMed Central PMCID: PMC6252083. 30415748 PMC6252083

[11] Hille B. Ion channels of excitable membranes 3rd ed. Sunderland, MA, USA: Sinauer Associated Inc; 2001. p. 1–22.

[12] Malcolm HR, Maurer JA. The mechanosensitive channel of small conductance (MscS) superfamily: not just mechanosensitive channels anymore. Chembiochem. 2012;13(14):2037–43. Epub 2012/08/24. doi: 10.1002/cbic.201200410 . 22915507

[13] Wo ZG, Oswald RE. Unraveling the modular design of glutamate-gated ion channels. Trends Neurosci. 1995;18(4):161–168. doi: 10.1016/0166-2236(95)93895-5 . 7539962

[14] Alexander SPH, Mathie A, Peters JA, Veale EL, Striessnig J, Kelly E, et al. THE CONCISE GUIDE TO PHARMACOLOGY 2019/20: Ion channels. Br J Pharmacol. 2019;176(S1):S142–S228. doi: 10.1111/bph.14749 31710715 PMC6844578

[15] Doyle DA, Morais Cabral J, Pfuetzner RA, Kuo A, Gulbis JM, Cohen SL, et al. The structure of the potassium channel: molecular basis of K+ conduction and selectivity. Science. 1998;280(5360):69–77. doi: 10.1126/science.280.5360.69 . 9525859

[16] Martinac B, Saimi Y, Kung C. Ion channels in microbes. Physiol Rev. 2008;88(4):1449–1490. doi: 10.1152/physrev.00005.2008 ; PubMed Central PMCID: PMC2579964. 18923187 PMC2579964

[17] Alexander SPH, Mathie A, Peters JA, Veale EL, Striessnig J, Kelly E, et al. THE CONCISE GUIDE TO PHARMACOLOGY 2019/20: Ion channels. Br J Pharmacol. 2019;176(Suppl 1):S142–s228. doi: 10.1111/bph.14749 ; PubMed Central PMCID: PMC6844578. 31710715 PMC6844578

[18] Gezelle J, Saggu G, Desai SA. Promises and Pitfalls of Parasite Patch-clamp. Trends Parasitol. 2021;37(5):414–29. Epub 20210224. doi: 10.1016/j.pt.2021.02.002 ; PubMed Central PMCID: PMC8049976. 33640269 PMC8049976

[19] Lee P, Ye Z, Van Dyke K, Kirk RG. X-ray microanalysis of Plasmodium falciparum and infected red blood cells: effects of qinghaosu and chloroquine on potassium, sodium, and phosphorus composition. Am J Trop Med Hyg. 1988;39(2):157–165. doi: 10.4269/ajtmh.1988.39.157 . 3044154

[20] Zhang M, Wang C, Otto TD, Oberstaller J, Liao X, Adapa SR, et al. Uncovering the essential genes of the human malaria parasite Plasmodium falciparum by saturation mutagenesis. Science. 2018;360(6388). doi: 10.1126/science.aap7847 ; PubMed Central PMCID: PMC6360947. 29724925 PMC6360947

[21] Otto TD, Böhme U, Jackson AP, Hunt M, Franke-Fayard B, Hoeijmakers WA, et al. A comprehensive evaluation of rodent malaria parasite genomes and gene expression. BMC Biol. 2014;12:86. Epub 20141030. doi: 10.1186/s12915-014-0086-0 ; PubMed Central PMCID: PMC4242472. 25359557 PMC4242472

[22] Kenthirapalan S, Waters AP, Matuschewski K, Kooij TW. Functional profiles of orphan membrane transporters in the life cycle of the malaria parasite. Nat Commun. 2016;7:10519. Epub 20160122. doi: 10.1038/ncomms10519 ; PubMed Central PMCID: PMC4736113. 26796412 PMC4736113

[23] Martin RE, Henry RI, Abbey JL, Clements JD, Kirk K. The ’permeome’ of the malaria parasite: an overview of the membrane transport proteins of Plasmodium falciparum. Genome Biol. 2005;6(3):R26. Epub 20050302. doi: 10.1186/gb-2005-6-3-r26 ; PubMed Central PMCID: PMC1088945. 15774027 PMC1088945

[24] Martin RE. The transportome of the malaria parasite. Biol Rev Camb Philos Soc. 2020;95(2):305–32. Epub 20191107. doi: 10.1111/brv.12565 . 31701663

[25] Kirk K, Lehane AM. Membrane transport in the malaria parasite and its host erythrocyte. Biochem J. 2014;457(1):1–18. doi: 10.1042/BJ20131007 . 24325549

[26] Martin RE, Ginsburg H, Kirk K. Membrane transport proteins of the malaria parasite. Mol Microbiol. 2009;74(3):519–28. Epub 20091001. doi: 10.1111/j.1365-2958.2009.06863.x . 19796339

[27] Kumari J, Rathore MS. Na(+)/K(+)-ATPase a Primary Membrane Transporter: An Overview and Recent Advances with Special Reference to Algae. J Membr Biol. 2020;253(3):191–204. Epub 20200519. doi: 10.1007/s00232-020-00119-0 . 32430620

[28] Sáez AG, Lozano E, Zaldívar-Riverón A. Evolutionary history of Na,K-ATPases and their osmoregulatory role. Genetica. 2009;136(3):479–90. Epub 20090213. doi: 10.1007/s10709-009-9356-0 . 19214758

[29] Rocco-Machado N, Cosentino-Gomes D, Meyer-Fernandes JR. Modulation of Na+/K+ ATPase Activity by Hydrogen Peroxide Generated through Heme in L. amazonensis. PLoS ONE. 2015;10(6):e0129604. Epub 20150612. doi: 10.1371/journal.pone.0129604 ; PubMed Central PMCID: PMC4466535. 26070143 PMC4466535

[30] Van der Heyden N, Docampo R. Significant differences between procyclic and bloodstream forms of Trypanosoma brucei in the maintenance of their plasma membrane potential. J Eukaryot Microbiol. 2002;49(5):407–413. doi: 10.1111/j.1550-7408.2002.tb00220.x . 12425529

[31] Van Der Heyden N, Docampo R. Proton and sodium pumps regulate the plasma membrane potential of different stages of Trypanosoma cruzi. Mol Biochem Parasitol. 2002;120(1):127–139. doi: 10.1016/s0166-6851(01)00444-3 . 11849712

[32] Vieira L, Slotki I, Cabantchik ZI. Chloride conductive pathways which support electrogenic H+ pumping by Leishmania major promastigotes. J Biol Chem. 1995;270(10):5299–5304. doi: 10.1074/jbc.270.10.5299 . 7890641

[33] Moreno SN, Zhong L, Lu HG, Souza WD, Benchimol M. Vacuolar-type H+-ATPase regulates cytoplasmic pH in Toxoplasma gondii tachyzoites. Biochem J. 1998;330(Pt 2):853–60. doi: 10.1042/bj3300853 ; PubMed Central PMCID: PMC1219216. 9480901 PMC1219216

[34] Biagini GA, Lloyd D, Kirk K, Edwards MR. The membrane potential of Giardia intestinalis. FEMS Microbiol Lett. 2000;192(1):153–157. doi: 10.1111/j.1574-6968.2000.tb09374.x . 11040444

[35] Allen RJ, Kirk K. The membrane potential of the intraerythrocytic malaria parasite Plasmodium falciparum. J Biol Chem. 2004;279(12):11264–72. Epub 20031120. doi: 10.1074/jbc.M311110200 . 14630911

[36] Henry RI, Cobbold SA, Allen RJ, Khan A, Hayward R, Lehane AM, et al. An acid-loading chloride transport pathway in the intraerythrocytic malaria parasite, Plasmodium falciparum. J Biol Chem. 2010;285(24):18615–26. Epub 20100323. doi: 10.1074/jbc.M110.120980 ; PubMed Central PMCID: PMC2881787. 20332090 PMC2881787

[37] Booth IR. Regulation of cytoplasmic pH in bacteria. Microbiol Rev. 1985;49(4):359–378. doi: 10.1128/mr.49.4.359-378.1985 ; PubMed Central PMCID: PMC373043. 3912654 PMC373043

[38] Cortázar TM, Hernández J, Echeverry MC, Camacho M. Role of the parasitophorous vacuole of murine macrophages infected with Leishmania amazonensis in molecule acquisition. Biomedica. 2006;26(Suppl 1):26–37. . 17361839

[39] Batista MF, Nájera CA, Meneghelli I, Bahia D. The Parasitic Intracellular Lifestyle of Trypanosomatids: Parasitophorous Vacuole Development and Survival. Front Cell Dev Biol. 2020;8:396. Epub 20200610. doi: 10.3389/fcell.2020.00396 ; PubMed Central PMCID: PMC7297907. 32587854 PMC7297907

[40] Kollien AH, Grospietsch T, Kleffmann T, Zerbst-Boroffka I, Schaub GA. Ionic composition of the rectal contents and excreta of the reduviid bug Triatoma infestans. J Insect Physiol 2001;47(7):739–47. Epub 2001/05/18. doi: 10.1016/s0022-1910(00)00170-0 . 11356421

[41] Contreras VT, Araujo-Jorge TC, Bonaldo MC, Thomaz N, Barbosa HS, Meirelles Mde N, et al. Biological aspects of the Dm 28c clone of Trypanosoma cruzi after metacyclogenesis in chemically defined media. Mem Inst Oswaldo Cruz. 1988;83(1):123–133. doi: 10.1590/s0074-02761988000100016 . 3074237

[42] Tomlinson S, Vandekerckhove F, Frevert U, Nussenzweig V. The induction of Trypanosoma cruzi trypomastigote to amastigote transformation by low pH. Parasitology. 1995;110(Pt 5):547–554. doi: 10.1017/s0031182000065264 . 7541124

[43] Shaw S, Knüsel S, Abbühl D, Naguleswaran A, Etzensperger R, Benninger M, et al. Cyclic AMP signalling and glucose metabolism mediate pH taxis by African trypanosomes. Nat Commun. 2022;13(1):603. Epub 20220201. doi: 10.1038/s41467-022-28293-w ; PubMed Central PMCID: PMC8807625. 35105902 PMC8807625

[44] Vanderheyden N, Benaim G, Docampo R. The role of a H(+)-ATPase in the regulation of cytoplasmic pH in Trypanosoma cruzi epimastigotes. Biochem J 1996;318(Pt 1):103–9. doi: 10.1042/bj3180103 ; PubMed Central PMCID: PMC1217594. 8761458 PMC1217594

[45] Van Der Heyden N, Docampo R. Intracellular pH in mammalian stages of Trypanosoma cruzi is K+-dependent and regulated by H+-ATPases. Mol Biochem Parasitol. 2000;105(2):237–251. doi: 10.1016/s0166-6851(99)00184-x . 10693746

[46] Yamashita N, Hagiwara S. Membrane depolarization and intracellular Ca2+ increase caused by high external Ca2+ in a rat calcitonin-secreting cell line. J Physiol. 1990;431:243–267. doi: 10.1113/jphysiol.1990.sp018329 ; PubMed Central PMCID: PMC1181773. 1712840 PMC1181773

[47] Catterall WA. Structure and regulation of voltage-gated Ca2+ channels. Annu Rev Cell Dev Biol. 2000;16:521–555. doi: 10.1146/annurev.cellbio.16.1.521 . 11031246

[48] Moreno SN, Silva J, Vercesi AE, Docampo R. Cytosolic-free calcium elevation in Trypanosoma cruzi is required for cell invasion. J Exp Med. 1994;180(4):1535–1540. doi: 10.1084/jem.180.4.1535 ; PubMed Central PMCID: PMC2191711. 7931085 PMC2191711

[49] Lovett JL, Marchesini N, Moreno SN, Sibley LD. Toxoplasma gondii microneme secretion involves intracellular Ca(2+) release from inositol 1,4,5-triphosphate (IP(3))/ryanodine-sensitive stores. J Biol Chem. 2002;277(29):25870–6. Epub 20020513. doi: 10.1074/jbc.M202553200 . 12011085

[50] Wetzel DM, Chen LA, Ruiz FA, Moreno SN, Sibley LD. Calcium-mediated protein secretion potentiates motility in Toxoplasma gondii. J Cell Sci. 2004;117(Pt 24):5739–48. Epub 20041026. doi: 10.1242/jcs.01495 . 15507483

[51] LaFavers KA, Márquez-Nogueras KM, Coppens I, Moreno SNJ, Arrizabalaga G. A novel dense granule protein, GRA41, regulates timing of egress and calcium sensitivity in Toxoplasma gondii. Cell Microbiol. 2017;19(9). Epub 20170517. doi: 10.1111/cmi.12749 ; PubMed Central PMCID: PMC5787377. 28436089 PMC5787377

[52] Márquez-Nogueras KM, Hortua Triana MA, Chasen NM, Kuo IY, Moreno SN. Calcium signaling through a transient receptor channel is important for Toxoplasma gondii growth. elife. 2021;10. Epub 20210609. doi: 10.7554/eLife.63417 ; PubMed Central PMCID: PMC8216714. 34106044 PMC8216714

[53] Stasic AJ, Dykes EJ, Cordeiro CD, Vella SA, Fazli MS, Quinn S, et al. Ca(2+) entry at the plasma membrane and uptake by acidic stores is regulated by the activity of the V-H(+) -ATPase in Toxoplasma gondii. Mol Microbiol. 2021;115(5):1054–68. Epub 20210419. doi: 10.1111/mmi.14722 ; PubMed Central PMCID: PMC9142151. 33793004 PMC9142151

[54] Balestra AC, Koussis K, Klages N, Howell SA, Flynn HR, Bantscheff M, et al. Ca(2+) signals critical for egress and gametogenesis in malaria parasites depend on a multipass membrane protein that interacts with PKG. Sci Adv. 2021;7(13). Epub 20210324. doi: 10.1126/sciadv.abe5396 ; PubMed Central PMCID: PMC7990342. 33762339 PMC7990342

[55] Lourido S, Moreno SN. The calcium signaling toolkit of the Apicomplexan parasites Toxoplasma gondii and Plasmodium spp. Cell Calcium. 2015;57(3):186–93. Epub 20141231. doi: 10.1016/j.ceca.2014.12.010 ; PubMed Central PMCID: PMC4428288. 25605521 PMC4428288

[56] Zhao H, Pan X. Mitochondrial Ca(2+) and cell cycle regulation. Int Rev Cell Mol Biol. 2021;362:171–207. Epub 20210409. doi: 10.1016/bs.ircmb.2021.02.015 . 34253295

[57] Li ZH, King TP, Ayong L, Asady B, Cai X, Rahman T, et al. A plastid two-pore channel essential for inter-organelle communication and growth of Toxoplasma gondii. Nat Commun. 2021;12(1):5802. Epub 20211004. doi: 10.1038/s41467-021-25987-5 ; PubMed Central PMCID: PMC8490419. 34608145 PMC8490419

[58] Rathore S, Datta G, Kaur I, Malhotra P, Mohmmed A. Disruption of cellular homeostasis induces organelle stress and triggers apoptosis like cell-death pathways in malaria parasite. Cell Death Dis. 2015;6(7):e1803. Epub 20150702. doi: 10.1038/cddis.2015.142 ; PubMed Central PMCID: PMC4650714. 26136076 PMC4650714

[59] Sanchez MA, Tran KD, Valli J, Hobbs S, Johnson E, Gluenz E, et al. KHARON Is an Essential Cytoskeletal Protein Involved in the Trafficking of Flagellar Membrane Proteins and Cell Division in African Trypanosomes. J Biol Chem. 2016;291(38):19760–73. Epub 20160803. doi: 10.1074/jbc.M116.739235 ; PubMed Central PMCID: PMC5025667. 27489106 PMC5025667

[60] Sen N, Das BB, Ganguly A, Mukherjee T, Bandyopadhyay S, Majumder HK. Camptothecin-induced imbalance in intracellular cation homeostasis regulates programmed cell death in unicellular hemoflagellate Leishmania donovani. J Biol Chem. 2004;279(50):52366–75. Epub 20040908. doi: 10.1074/jbc.M406705200 . 15355995

[61] Damasceno FS, Barisón MJ, Pral EM, Paes LS, Silber AM. Memantine, an antagonist of the NMDA glutamate receptor, affects cell proliferation, differentiation and the intracellular cycle and induces apoptosis in Trypanosoma cruzi. PLoS Negl Trop Dis. 2014;8(2):e2717. Epub 20140227. doi: 10.1371/journal.pntd.0002717 ; PubMed Central PMCID: PMC3937314. 24587468 PMC3937314

[62] Bosia A, Ghigo D, Turrini F, Nissani E, Pescarmona GP, Ginsburg H. Kinetic characterization of Na+/H+ antiport of Plasmodium falciparum membrane. J Cell Physiol. 1993;154(3):527–534. doi: 10.1002/jcp.1041540311 . 8382209

[63] Fraser-L’Hostis C, Defrise-Quertain F, Coral D, Deshusses J. Regulation of the intracellular pH in the protozoan parasite Trypanosoma brucei brucei. Biol Chem. 1997;378(9):1039–1046. doi: 10.1515/bchm.1997.378.9.1039 . 9348114

[64] Francia ME, Wicher S, Pace DA, Sullivan J, Moreno SN, Arrizabalaga G. A Toxoplasma gondii protein with homology to intracellular type Na^+^/H^+^ exchangers is important for osmoregulation and invasion. Exp Cell Res. 2011;317(10):1382–96. Epub 20110409. doi: 10.1016/j.yexcr.2011.03.020 ; PubMed Central PMCID: PMC3096714. 21501607 PMC3096714

[65] Krishna S, Woodrow C, Webb R, Penny J, Takeyasu K, Kimura M, et al. Expression and functional characterization of a Plasmodium falciparum Ca2+-ATPase (PfATP4) belonging to a subclass unique to apicomplexan organisms. J Biol Chem. 2001;276(14):10782–7. Epub 20010105. doi: 10.1074/jbc.M010554200 . 11145964

[66] Rosling JEO, Ridgway MC, Summers RL, Kirk K, Lehane AM. Biochemical characterization and chemical inhibition of PfATP4-associated Na(+)-ATPase activity in Plasmodium falciparum membranes. J Biol Chem. 2018;293(34):13327–37. Epub 20180709. doi: 10.1074/jbc.RA118.003640 ; PubMed Central PMCID: PMC6109929. 29986883 PMC6109929

[67] Spillman NJ, Allen RJ, McNamara CW, Yeung BK, Winzeler EA, Diagana TT, et al. Na(+) regulation in the malaria parasite Plasmodium falciparum involves the cation ATPase PfATP4 and is a target of the spiroindolone antimalarials. Cell Host Microbe. 2013;13(2):227–237. doi: 10.1016/j.chom.2012.12.006 ; PubMed Central PMCID: PMC3574224. 23414762 PMC3574224

[68] Dick CF, Meyer-Fernandes JR, Vieyra A. The Functioning of Na(+)-ATPases from Protozoan Parasites: Are These Pumps Targets for Antiparasitic Drugs? Cell. 2020;9(10). Epub 20201002. doi: 10.3390/cells9102225 ; PubMed Central PMCID: PMC7600311. 33023071 PMC7600311

[69] Ullah I, Sharma R, Mete A, Biagini GA, Wetzel DM, Horrocks PD. The relative rate of kill of the MMV Malaria Box compounds provides links to the mode of antimalarial action and highlights scaffolds of medicinal chemistry interest. J Antimicrob Chemother. 2020;75(2):362–370. doi: 10.1093/jac/dkz443 ; PubMed Central PMCID: PMC8204706. 31665424 PMC8204706

[70] Lehane AM, Dennis ASM, Bray KO, Li D, Rajendran E, McCoy JM, et al. Characterization of the ATP4 ion pump in Toxoplasma gondii. J Biol Chem. 2019;294(14):5720–34. Epub 20190205. doi: 10.1074/jbc.RA118.006706 ; PubMed Central PMCID: PMC6462519. 30723156 PMC6462519

[71] Caruso-Neves C, Einicker-Lamas M, Chagas C, Oliveira MM, Vieyra A, Lopes AG. Ouabain-insensitive Na(+)-ATPase activity in Trypanosoma cruzi epimastigotes. Z Naturforsch C J Biosci. 1999;54(1–2):100–104. . 10097410

[72] Iizumi K, Mikami Y, Hashimoto M, Nara T, Hara Y, Aoki T. Molecular cloning and characterization of ouabain-insensitive Na(+)-ATPase in the parasitic protist, Trypanosoma cruzi. Biochim Biophys Acta. 2006;1758(6):738–46. Epub 20060516. doi: 10.1016/j.bbamem.2006.04.025 . 16797482

[73] Stiles JK, Kucerova Z, Sarfo B, Meade CA, Thompson W, Shah P, et al. Identification of surface-membrane P-type ATPases resembling fungal K(+)- and Na(+)-ATPases, in Trypanosoma brucei, Trypanosoma cruzi and Leishmania donovani. Ann Trop Med Parasitol. 2003;97(4):351–366. doi: 10.1179/000349803235002362 . 12831521

[74] Benito B, Garciadeblás B, Rodrı G-NA. Potassium- or sodium-efflux ATPase, a key enzyme in the evolution of fungi. Microbiology (Reading). 2002;148(Pt 4):933–941. doi: 10.1099/00221287-148-4-933 . 11932440

[75] de Almeida-Amaral EE, Caruso-Neves C, Pires VM, Meyer-Fernandes JR. Leishmania amazonensis: characterization of an ouabain-insensitive Na+-ATPase activity. Exp Parasitol. 2008;118(2):165–71. Epub 20070803. doi: 10.1016/j.exppara.2007.07.001 . 17825292

[76] Jimenez V, Docampo R. Molecular and electrophysiological characterization of a novel cation channel of Trypanosoma cruzi. PLoS Pathog. 2012;8(6):e1002750. Epub 20120607. doi: 10.1371/journal.ppat.1002750 ; PubMed Central PMCID: PMC3369953. 22685407 PMC3369953

[77] Bachmann M, Li W, Edwards MJ, Ahmad SA, Patel S, Szabo I, et al. Voltage-Gated Potassium Channels as Regulators of Cell Death. Front Cell Dev Biol. 2020;8:611853. Epub 20201214. doi: 10.3389/fcell.2020.611853 ; PubMed Central PMCID: PMC7767978. 33381507 PMC7767978

[78] Marchesini N, Docampo R. A plasma membrane P-type H(+)-ATPase regulates intracellular pH in Leishmania mexicana amazonensis. Mol Biochem Parasitol. 2002;119(2):225–236. doi: 10.1016/s0166-6851(01)00419-4 . 11814574

[79] Prole DL, Marrion NV. Identification of putative potassium channel homologues in pathogenic protozoa. PLoS ONE. 2012;7(2):e32264. Epub 20120221. doi: 10.1371/journal.pone.0032264 ; PubMed Central PMCID: PMC3283738. 22363819 PMC3283738

[80] Jimenez V, Henriquez M, Galanti N, Riquelme G. Electrophysiological characterization of potassium conductive pathways in Trypanosoma cruzi. J Cell Biochem. 2011;112(4):1093–1102. doi: 10.1002/jcb.23023 . 21308738

[81] MacKinnon R, Cohen SL, Kuo A, Lee A, Chait BT. Structural conservation in prokaryotic and eukaryotic potassium channels. Science. 1998;280(5360):106–109. doi: 10.1126/science.280.5360.106 . 9525854

[82] Barrera P, Skorka C, Boktor M, Dave N, Jimenez V. A Novel Calcium-Activated Potassium Channel Controls Membrane Potential and Intracellular pH in Trypanosoma cruzi. Front Cell Infect Microbiol. 2019;9:464. Epub 20200115. doi: 10.3389/fcimb.2019.00464 ; PubMed Central PMCID: PMC6974456. 32010643 PMC6974456

[83] Steinmann ME, González-Salgado A, Bütikofer P, Mäser P, Sigel E. A heteromeric potassium channel involved in the modulation of the plasma membrane potential is essential for the survival of African trypanosomes. FASEB J. 2015;29(8):3228–37. Epub 20150413. doi: 10.1096/fj.15-271353 . 25868728

[84] Paul A, Mubashra, Singh S. Identification of a novel calcium activated potassium channel from Leishmania donovani and in silico predictions of its antigenic features. Acta Trop. 2021;220:105922. Epub 20210418. doi: 10.1016/j.actatropica.2021.105922 . 33878308

[85] Sudhandiran G, Shaha C. Antimonial-induced increase in intracellular Ca2+ through non-selective cation channels in the host and the parasite is responsible for apoptosis of intracellular Leishmania donovani amastigotes. J Biol Chem. 2003;278(27):25120–32. Epub 20030421. doi: 10.1074/jbc.M301975200 . 12707265

[86] Mukherjee SB, Das M, Sudhandiran G, Shaha C. Increase in cytosolic Ca2+ levels through the activation of non-selective cation channels induced by oxidative stress causes mitochondrial depolarization leading to apoptosis-like death in Leishmania donovani promastigotes. J Biol Chem. 2002;277(27):24717–27. Epub 20020430. doi: 10.1074/jbc.M201961200 . 11983701

[87] Waller KL, McBride SM, Kim K, McDonald TV. Characterization of two putative potassium channels in Plasmodium falciparum. Malar J. 2008;7:19. Epub 20080124. doi: 10.1186/1475-2875-7-19 ; PubMed Central PMCID: PMC2263067. 18218136 PMC2263067

[88] Molbaek K, Tejada M, Ricke CH, Scharff-Poulsen P, Ellekvist P, Helix-Nielsen C, et al. Purification and initial characterization of Plasmodium falciparum K(+) channels, PfKch1 and PfKch2 produced in Saccharomyces cerevisiae. Microb Cell Factories. 2020;19(1):183. Epub 20200921. doi: 10.1186/s12934-020-01437-7 ; PubMed Central PMCID: PMC7507820. 32957994 PMC7507820

[89] Ellekvist P, Mlambo G, Kumar N, Klaerke DA. Functional characterization of malaria parasites deficient in the K(+) channel Kch2. Biochem Biophys Res Commun. 2017;493(1):690–6. Epub 20170830. doi: 10.1016/j.bbrc.2017.08.128 ; PubMed Central PMCID: PMC5636674. 28864420 PMC5636674

[90] Ellekvist P, Maciel J, Mlambo G, Ricke CH, Colding H, Klaerke DA, et al. Critical role of a K+ channel in Plasmodium berghei transmission revealed by targeted gene disruption. Proc Natl Acad Sci U S A. 2008;105(17):6398–6402. doi: 10.1073/pnas.0802384105 18434537 PMC2359770

[91] Nugues C, Helassa N, Haynes LP. Mitosis, Focus on Calcium. Front Physiol. 2022;13:951979. Epub 20220617. doi: 10.3389/fphys.2022.951979 ; PubMed Central PMCID: PMC9247304. 35784871 PMC9247304

[92] Curcic S, Schober R, Schindl R, Groschner K. TRPC-mediated Ca(2+) signaling and control of cellular functions. Semin Cell Dev Biol. 2019;94:28–39. Epub 20190302. doi: 10.1016/j.semcdb.2019.02.001 . 30738858

[93] Kito H, Ohya S. Role of K(+) and Ca(2+)-Permeable Channels in Osteoblast Functions. Int J Mol Sci. 2021;22(19). Epub 20210928. doi: 10.3390/ijms221910459 ; PubMed Central PMCID: PMC8509041. 34638799 PMC8509041

[94] Sukumaran P, Nascimento Da Conceicao V, Sun Y, Ahamad N, Saraiva LR, Selvaraj S, et al. Calcium Signaling Regulates Autophagy and Apoptosis. Cell. 2021;10(8). Epub 20210818. doi: 10.3390/cells10082125 ; PubMed Central PMCID: PMC8394685. 34440894 PMC8394685

[95] Adiga D, Radhakrishnan R, Chakrabarty S, Kumar P, Kabekkodu SP. The Role of Calcium Signaling in Regulation of Epithelial-Mesenchymal Transition. Cells Tissues Organs. 2022;211(2):134–56. Epub 20201214. doi: 10.1159/000512277 . 33316804

[96] Wei J, Deng Y, Ye J, Luo Y, Weng J, He Q, et al. Store-operated Ca(2+) entry as a key oncogenic Ca(2+) signaling driving tumor invasion-metastasis cascade and its translational potential. Cancer Lett. 2021;516:64–72. Epub 20210602. doi: 10.1016/j.canlet.2021.05.036 . 34089807

[97] Mata-Martínez E, Sánchez-Cárdenas C, Chávez JC, Guerrero A, Treviño CL, Corkidi G, et al. Role of calcium oscillations in sperm physiology. Biosystems. 2021;209:104524. Epub 20210826. doi: 10.1016/j.biosystems.2021.104524 . 34453988

[98] Lu H-G, Zhong L, Chang K-P, Docampo R. Intracellular Ca2+ Pool Content and Signaling and Expression of a Calcium Pump Are Linked to Virulence in Leishmania mexicana amazonesis Amastigotes*. J Biol Chem. 1997;272(14):9464–73. doi: 10.1074/jbc.272.14.9464 9083086

[99] Bullen HE, Bisio H, Soldati-Favre D. The triumvirate of signaling molecules controlling Toxoplasma microneme exocytosis: Cyclic GMP, calcium, and phosphatidic acid. PLoS Pathog. 2019;15(5):e1007670. Epub 20190523. doi: 10.1371/journal.ppat.1007670 ; PubMed Central PMCID: PMC6532924. 31121005 PMC6532924

[100] Fu Y, Brown KM, Jones NG, Moreno SN, Sibley LD. Toxoplasma bradyzoites exhibit physiological plasticity of calcium and energy stores controlling motility and egress. elife. 2021;10. Epub 20211203. doi: 10.7554/eLife.73011 ; PubMed Central PMCID: PMC8683080. 34860156 PMC8683080

[101] Kafsack BF, Pena JD, Coppens I, Ravindran S, Boothroyd JC, Carruthers VB. Rapid membrane disruption by a perforin-like protein facilitates parasite exit from host cells. Science. 2009;323(5913):530–3. Epub 20081218. doi: 10.1126/science.1165740 ; PubMed Central PMCID: PMC2662845. 19095897 PMC2662845

[102] Dvorin JD, Martyn DC, Patel SD, Grimley JS, Collins CR, Hopp CS, et al. A plant-like kinase in Plasmodium falciparum regulates parasite egress from erythrocytes. Science. 2010;328(5980):910–912. doi: 10.1126/science.1188191 ; PubMed Central PMCID: PMC3109083. 20466936 PMC3109083

[103] Glushakova S, Lizunov V, Blank PS, Melikov K, Humphrey G, Zimmerberg J. Cytoplasmic free Ca2+ is essential for multiple steps in malaria parasite egress from infected erythrocytes. Malar J. 2013;12:41. Epub 20130130. doi: 10.1186/1475-2875-12-41 ; PubMed Central PMCID: PMC3564835. 23363708 PMC3564835

[104] Apolis L, Olivas J, Srinivasan P, Kushwaha AK, Desai SA. Multiple genetic loci define Ca(++) utilization by bloodstream malaria parasites. BMC Genomics. 2019;20(1):47. Epub 20190116. doi: 10.1186/s12864-018-5418-y ; PubMed Central PMCID: PMC6335690. 30651090 PMC6335690

[105] Benaim G, García-Marchán Y, Reyes C, Uzcanga G, Figarella K. Identification of a sphingosine-sensitive Ca2+ channel in the plasma membrane of Leishmania mexicana. Biochem Biophys Res Commun. 2013;430(3):1091–6. Epub 20121219. doi: 10.1016/j.bbrc.2012.12.033 . 23261440

[106] Borges-Pereira L, Thomas SJ, Dos Anjos ESAL, Bartlett PJ, Thomas AP, Garcia CRS. The genetic Ca(2+) sensor GCaMP3 reveals multiple Ca(2+) stores differentially coupled to Ca(2+) entry in the human malaria parasite Plasmodium falciparum. J Biol Chem. 2020;295(44):14998–5012. Epub 20200826. doi: 10.1074/jbc.RA120.014906 ; PubMed Central PMCID: PMC7606694. 32848018 PMC7606694

[107] Budu A, Gomes MM, Melo PM, El Chamy Maluf S, Bagnaresi P, Azevedo MF, et al. Calmidazolium evokes high calcium fluctuations in Plasmodium falciparum. Cell Signal. 2016;28(3):125–35. Epub 20151210. doi: 10.1016/j.cellsig.2015.12.003 . 26689736

[108] Pérez-Gordones MC, Ramírez-Iglesias JR, Benaim G, Mendoza M. A store-operated Ca(2+)-entry in Trypanosoma equiperdum: Physiological evidences of its presence. Mol Biochem Parasitol. 2021;244:111394. Epub 20210701. doi: 10.1016/j.molbiopara.2021.111394 . 34216677

[109] Manchola Varón NC, Dos Santos G, Colli W, Alves MJM. Interaction With the Extracellular Matrix Triggers Calcium Signaling in Trypanosoma cruzi Prior to Cell Invasion. Front Cell Infect Microbiol. 2021;11:731372. Epub 20211004. doi: 10.3389/fcimb.2021.731372 ; PubMed Central PMCID: PMC8521164. 34671568 PMC8521164

[110] Rodriguez-Duran J, Pinto-Martinez A, Castillo C, Benaim G. Identification and electrophysiological properties of a sphingosine-dependent plasma membrane Ca(2+) channel in Trypanosoma cruzi. FEBS J. 2019;286(19):3909–25. Epub 20190628. doi: 10.1111/febs.14947 . 31162791

[111] Dave N, Cetiner U, Arroyo D, Fonbuena J, Tiwari M, Barrera P, et al. A novel mechanosensitive channel controls osmoregulation, differentiation, and infectivity in Trypanosoma cruzi. elife. 2021;10. Epub 20210702. doi: 10.7554/eLife.67449 ; PubMed Central PMCID: PMC8282336. 34212856 PMC8282336

[112] Pinto-Martinez AK, Rodriguez-Durán J, Serrano-Martin X, Hernandez-Rodriguez V, Benaim G. Mechanism of Action of Miltefosine on Leishmania donovani Involves the Impairment of Acidocalcisome Function and the Activation of the Sphingosine-Dependent Plasma Membrane Ca(2+) Channel Antimicrob Agents Chemother. 2018;62(1). Epub 20171221. doi: 10.1128/aac.01614-17 ; PubMed Central PMCID: PMC5740361. 29061745 PMC5740361

[113] Prole DL, Taylor CW. Identification of intracellular and plasma membrane calcium channel homologues in pathogenic parasites. PLoS ONE. 2011;6(10):e26218. Epub 20111014. doi: 10.1371/journal.pone.0026218 ; PubMed Central PMCID: PMC3194816. 22022573 PMC3194816

[114] Oberholzer M, Langousis G, Nguyen HT, Saada EA, Shimogawa MM, Jonsson ZO, et al. Independent analysis of the flagellum surface and matrix proteomes provides insight into flagellum signaling in mammalian-infectious Trypanosoma brucei. Mol Cell Proteomics. 2011;10(10):M111.010538. Epub 20110619. doi: 10.1074/mcp.M111.010538 ; PubMed Central PMCID: PMC3205874. 21685506 PMC3205874

[115] Kawamoto F, Alejo-Blanco R, Fleck SL, Kawamoto Y, Sinden RE. Possible roles of Ca2+ and cGMP as mediators of the exflagellation of Plasmodium berghei and Plasmodium falciparum. Mol Biochem Parasitol. 1990;42(1):101–108. doi: 10.1016/0166-6851(90)90117-5 . 2172816

[116] Okada Y, Okada T, Sato-Numata K, Islam MR, Ando-Akatsuka Y, Numata T, et al. Cell Volume-Activated and Volume-Correlated Anion Channels in Mammalian Cells: Their Biophysical, Molecular, and Pharmacological Properties. Pharmacol Rev. 2019;71(1):49–88. doi: 10.1124/pr.118.015917 . 30573636

[117] Okada Y, Sabirov RZ, Sato-Numata K, Numata T. Cell Death Induction and Protection by Activation of Ubiquitously Expressed Anion/Cation Channels. Part 1: Roles of VSOR/VRAC in Cell Volume Regulation, Release of Double-Edged Signals and Apoptotic/Necrotic Cell Death. Front Cell Dev Biol. 2020;8:614040. Epub 20210112. doi: 10.3389/fcell.2020.614040 ; PubMed Central PMCID: PMC7835517. 33511120 PMC7835517

[118] Kim HJ, Lee PC, Hong JH. Chloride Channels and Transporters: Roles beyond Classical Cellular Homeostatic pH or Ion Balance in Cancers. Cancers (Basel). 2022;14(4). Epub 20220209. doi: 10.3390/cancers14040856 ; PubMed Central PMCID: PMC8870652. 35205604 PMC8870652

[119] Farinha CM, Callebaut I. Molecular mechanisms of cystic fibrosis—how mutations lead to misfunction and guide therapy. Biosci Rep. 2022;42(7). doi: 10.1042/bsr20212006 ; PubMed Central PMCID: PMC9251585. 35707985 PMC9251585

[120] Shah VS, Chivukula RR, Lin B, Waghray A, Rajagopal J. Cystic Fibrosis and the Cells of the Airway Epithelium: What Are Ionocytes and What Do They Do? Annu Rev Pathol. 2022;17:23–46. Epub 20210826. doi: 10.1146/annurev-pathol-042420-094031 . 34437820 PMC10837786

[121] Salas-Casas A, Ponce-Balderas A, García-Pérez RM, Cortés-Reynosa P, Gamba G, Orozco E, et al. Identification and functional characterization of EhClC-A, an Entamoeba histolytica ClC chloride channel located at plasma membrane. Mol Microbiol. 2006;59(4):1249–1261. doi: 10.1111/j.1365-2958.2006.05023.x . 16430698

[122] Moreno-Galindo EG, Rodríguez-Elías JC, Ramírez-Herrera MA, Sánchez-Chapula JA, Navarro-Polanco RA. The principal conductance in Giardia lamblia trophozoites possesses functional properties similar to the mammalian ClC-2 current. Pflugers Arch. 2014;466(5):915–24. Epub 20130917. doi: 10.1007/s00424-013-1350-9 . 24043571

[123] Lagos ML, Moran O, Camacho M. Leishmania amazonensis: Anionic currents expressed in oocytes upon microinjection of mRNA from the parasite. Exp Parasitol. 2007;116(2):163–70. Epub 20070105. doi: 10.1016/j.exppara.2006.12.010 . 17328895

[124] Ponte-Sucre A, Campos Y, Fernandez M, Moll H, Mendoza-León A. Leishmania sp.: growth and survival are impaired by ion channel blockers. Exp Parasitol. 1998;88(1):11–19. doi: 10.1006/expr.1998.4200 . 9501844

[125] Denker BM, Smith BL, Kuhajda FP, Agre P. Identification, purification, and partial characterization of a novel Mr 28,000 integral membrane protein from erythrocytes and renal tubules. J Biol Chem. 1988;263(30):15634–15642. . 3049610

[126] Agre P, Kozono D. Aquaporin water channels: molecular mechanisms for human diseases. FEBS Lett. 2003;555(1):72–78. doi: 10.1016/s0014-5793(03)01083-4 . 14630322

[127] Ishibashi K, Tanaka Y, Morishita Y. The role of mammalian superaquaporins inside the cell: An update. Biochim Biophys Acta Biomembr. 2021;1863(7):183617. doi: 10.1016/j.bbamem.2021.183617 33811846

[128] Pavlovic-Djuranovic S, Schultz JE, Beitz E. A single aquaporin gene encodes a water/glycerol/urea facilitator in Toxoplasma gondii with similarity to plant tonoplast intrinsic proteins. FEBS Lett. 2003;555(3):500–504. doi: 10.1016/s0014-5793(03)01313-9 . 14675763

[129] Hansen M, Kun JF, Schultz JE, Beitz E. A single, bi-functional aquaglyceroporin in blood-stage Plasmodium falciparum malaria parasites. J Biol Chem. 2002;277(7):4874–82. Epub 20011129. doi: 10.1074/jbc.M110683200 . 11729204

[130] Beitz E. Aquaporins from pathogenic protozoan parasites: structure, function and potential for chemotherapy. Biol Cell. 2005;97(6):373–383. doi: 10.1042/BC20040095 . 15901246

[131] Figarella K, Uzcategui NL, Zhou Y, LeFurgey A, Ouellette M, Bhattacharjee H, et al. Biochemical characterization of Leishmania major aquaglyceroporin LmAQP1: possible role in volume regulation and osmotaxis. Mol Microbiol. 2007;65(4):1006–17. Epub 20070719. doi: 10.1111/j.1365-2958.2007.05845.x . 17640270

[132] Gourbal B, Sonuc N, Bhattacharjee H, Legare D, Sundar S, Ouellette M, et al. Drug uptake and modulation of drug resistance in Leishmania by an aquaglyceroporin. J Biol Chem. 2004;279(30):31010–7. Epub 20040511. doi: 10.1074/jbc.M403959200 . 15138256

[133] Biyani N, Mandal S, Seth C, Saint M, Natarajan K, Ghosh I, et al. Characterization of Leishmania donovani aquaporins shows presence of subcellular aquaporins similar to tonoplast intrinsic proteins of plants. PLoS ONE. 2011;6(9):e24820. Epub 20110928. doi: 10.1371/journal.pone.0024820 ; PubMed Central PMCID: PMC3182166. 21969862 PMC3182166

[134] Von Bülow J, Beitz E. Number and regulation of protozoan aquaporins reflect environmental complexity. Biol Bull. 2015;229(1):38–46. doi: 10.1086/BBLv229n1p38 . 26338868

[135] Montalvetti A, Rohloff P, Docampo R. A functional aquaporin co-localizes with the vacuolar proton pyrophosphatase to acidocalcisomes and the contractile vacuole complex of Trypanosoma cruzi. J Biol Chem. 2004;279(37):38673–82. Epub 20040712. doi: 10.1074/jbc.M406304200 . 15252016

[136] Rohloff P, Montalvetti A, Docampo R. Acidocalcisomes and the contractile vacuole complex are involved in osmoregulation in Trypanosoma cruzi. J Biol Chem. 2004;279(50):52270–81. Epub 20041004. doi: 10.1074/jbc.M410372200 . 15466463

[137] Bassarak B, Uzcátegui NL, Schönfeld C, Duszenko M. Functional characterization of three aquaglyceroporins from Trypanosoma brucei in osmoregulation and glycerol transport. Cell Physiol Biochem. 2011;27(3–4):411–20. Epub 20110401. doi: 10.1159/000327968 . 21471730

[138] Uzcategui NL, Szallies A, Pavlovic-Djuranovic S, Palmada M, Figarella K, Boehmer C, et al. Cloning, heterologous expression, and characterization of three aquaglyceroporins from Trypanosoma brucei. J Biol Chem. 2004;279(41):42669–76. Epub 20040804. doi: 10.1074/jbc.M404518200 . 15294911

[139] Tesan FC, Lorenzo R, Alleva K, Fox AR. AQPX-cluster aquaporins and aquaglyceroporins are asymmetrically distributed in trypanosomes. Commun Biol. 2021;4(1):953. Epub 20210810. doi: 10.1038/s42003-021-02472-9 ; PubMed Central PMCID: PMC8355241. 34376792 PMC8355241

[140] Munday JC, Eze AA, Baker N, Glover L, Clucas C, Aguinaga Andrés D, et al. Trypanosoma brucei aquaglyceroporin 2 is a high-affinity transporter for pentamidine and melaminophenyl arsenic drugs and the main genetic determinant of resistance to these drugs. J Antimicrob Chemother. 2014;69(3):651–63. Epub 20131113. doi: 10.1093/jac/dkt442 ; PubMed Central PMCID: PMC3922157. 24235095 PMC3922157

[141] Mandal G, Mandal S, Sharma M, Charret KS, Papadopoulou B, Bhattacharjee H, et al. Species-specific antimonial sensitivity in Leishmania is driven by post-transcriptional regulation of AQP1. PLoS Negl Trop Dis. 2015;9(2):e0003500. Epub 20150225. doi: 10.1371/journal.pntd.0003500 ; PubMed Central PMCID: PMC4340957. 25714343 PMC4340957

[142] Baker N, Glover L, Munday JC, Aguinaga Andrés D, Barrett MP, de Koning HP, et al. Aquaglyceroporin 2 controls susceptibility to melarsoprol and pentamidine in African trypanosomes. Proc Natl Acad Sci U S A. 2012;109(27):10996–1001. Epub 20120618. doi: 10.1073/pnas.1202885109 ; PubMed Central PMCID: PMC3390834. 22711816 PMC3390834

[143] Posfai D, Maher SP, Roesch C, Vantaux A, Sylvester K, Péneau J, et al. Plasmodium vivax Liver and Blood Stages Recruit the Druggable Host Membrane Channel Aquaporin-3. Cell Chem Biol. 2020;27(6):719–27.e5. Epub 20200423. doi: 10.1016/j.chembiol.2020.03.009 ; PubMed Central PMCID: PMC7303948. 32330444 PMC7303948

[144] Posfai D, Sylvester K, Reddy A, Ganley JG, Wirth J, Cullen QE, et al. Plasmodium parasite exploits host aquaporin-3 during liver stage malaria infection. PLoS Pathog. 2018;14(5):e1007057. Epub 20180518. doi: 10.1371/journal.ppat.1007057 ; PubMed Central PMCID: PMC5979039. 29775485 PMC5979039

[145] Amanzougaghene N, Tajeri S, Yalaoui S, Lorthiois A, Soulard V, Gego A, et al. The Host Protein Aquaporin-9 is Required for Efficient Plasmodium falciparum Sporozoite Entry into Human Hepatocytes. Front Cell Infect Microbiol. 2021;11:704662. Epub 20210629. doi: 10.3389/fcimb.2021.704662 ; PubMed Central PMCID: PMC8276244. 34268141 PMC8276244

[146] Pernas L, Scorrano L. Mito-Morphosis: Mitochondrial Fusion, Fission, and Cristae Remodeling as Key Mediators of Cellular Function. Annu Rev Physiol. 2016;78:505–31. Epub 20151119. doi: 10.1146/annurev-physiol-021115-105011 . 26667075

[147] Glancy B, Kim Y, Katti P, Willingham TB. The Functional Impact of Mitochondrial Structure Across Subcellular Scales. Front Physiol. 2020;11:541040. Epub 20201111. doi: 10.3389/fphys.2020.541040 ; PubMed Central PMCID: PMC7686514. 33262702 PMC7686514

[148] Martijn J, Vosseberg J, Guy L, Offre P, Ettema TJG. Deep mitochondrial origin outside the sampled alphaproteobacteria. Nature. 2018;557(7703):101–5. Epub 20180425. doi: 10.1038/s41586-018-0059-5 . 29695865

[149] Käser S, Oeljeklaus S, Týč J, Vaughan S, Warscheid B, Schneider A. Outer membrane protein functions as integrator of protein import and DNA inheritance in mitochondria. Proc Natl Acad Sci U S A. 2016;113(31):E4467–75. Epub 20160719. doi: 10.1073/pnas.1605497113 ; PubMed Central PMCID: PMC4978248. 27436903 PMC4978248

[150] Hoppins S, Collins SR, Cassidy-Stone A, Hummel E, Devay RM, Lackner LL, et al. A mitochondrial-focused genetic interaction map reveals a scaffold-like complex required for inner membrane organization in mitochondria. J Cell Biol. 2011;195(2):323–40. Epub 20111010. doi: 10.1083/jcb.201107053 ; PubMed Central PMCID: PMC3198156. 21987634 PMC3198156

[151] Harner M, Körner C, Walther D, Mokranjac D, Kaesmacher J, Welsch U, et al. The mitochondrial contact site complex, a determinant of mitochondrial architecture. EMBO J. 2011;30(21):4356–70. Epub 20111018. doi: 10.1038/emboj.2011.379 ; PubMed Central PMCID: PMC3230385. 22009199 PMC3230385

[152] Ford HC, Allen WJ, Pereira GC, Liu X, Dillingham MS, Collinson I. Towards a molecular mechanism underlying mitochondrial protein import through the TOM and TIM23 complexes. elife. 2022;11. Epub 20220608. doi: 10.7554/eLife.75426 ; PubMed Central PMCID: PMC9255969. 35674314 PMC9255969

[153] Li X, Straub J, Medeiros TC, Mehra C, den Brave F, Peker E, et al. Mitochondria shed their outer membrane in response to infection-induced stress. Science. 2022;375(6577):eabi4343. Epub 20220114. doi: 10.1126/science.abi4343 . 35025629

[154] Brokatzky D, Häcker G. Mitochondria: intracellular sentinels of infections. Med Microbiol Immunol. 2022;211(4):161–72. Epub 20220705. doi: 10.1007/s00430-022-00742-9 ; PubMed Central PMCID: PMC9255486. 35790577 PMC9255486

[155] Medeiros TC, Mehra C, Pernas L. Contact and competition between mitochondria and microbes. Curr Opin Microbiol. 2021;63:189–94. Epub 20210816. doi: 10.1016/j.mib.2021.07.014 . 34411806

[156] Schnaufer A, Clark-Walker GD, Steinberg AG, Stuart K. The F1-ATP synthase complex in bloodstream stage trypanosomes has an unusual and essential function. EMBO J. 2005;24(23):4029–40. doi: 10.1038/sj.emboj.7600862 16270030 PMC1356303

[157] Brown SV, Hosking P, Li J, Williams N. ATP synthase is responsible for maintaining mitochondrial membrane potential in bloodstream form Trypanosoma brucei. Eukaryot Cell. 2006;5(1):45–53. doi: 10.1128/EC.5.1.45-53.2006 ; PubMed Central PMCID: PMC1360250. 16400167 PMC1360250

[158] Mühleip AW, Dewar CE, Schnaufer A, Kühlbrandt W, Davies KM. In situ structure of trypanosomal ATP synthase dimer reveals a unique arrangement of catalytic subunits. Proc Natl Acad Sci. 2017;114(5):992–997. doi: 10.1073/pnas.1612386114 28096380 PMC5293049

[159] Melo EJ, Attias M, De Souza W. The single mitochondrion of tachyzoites of Toxoplasma gondii. J Struct Biol. 2000;130(1):27–33. doi: 10.1006/jsbi.2000.4228 . 10806088

[160] van Dooren GG, Yeoh LM, Striepen B, McFadden GI. The Import of Proteins into the Mitochondrion of Toxoplasma gondii. J Biol Chem. 2016;291(37):19335–50. Epub 20160725. doi: 10.1074/jbc.M116.725069 ; PubMed Central PMCID: PMC5016674. 27458014 PMC5016674

[161] Stanway RR, Mueller N, Zobiak B, Graewe S, Froehlke U, Zessin PJ, et al. Organelle segregation into Plasmodium liver stage merozoites. Cell Microbiol. 2011;13(11):1768–82. Epub 20110831. doi: 10.1111/j.1462-5822.2011.01657.x . 21801293

[162] Evers F, Cabrera-Orefice A, Elurbe DM, Kea-Te Lindert M, Boltryk SD, Voss TS, et al. Composition and stage dynamics of mitochondrial complexes in Plasmodium falciparum. Nat Commun. 2021;12(1):3820. Epub 20210621. doi: 10.1038/s41467-021-23919-x ; PubMed Central PMCID: PMC8217502. 34155201 PMC8217502

[163] Putignani L. The unusual architecture and predicted function of the mitochondrion organelle in Cryptosporidium parvum and hominis species: the strong paradigm of the structure-function relationship. Parassitologia. 2005;47(2):217–225. . 16252476

[164] Putignani L, Tait A, Smith HV, Horner D, Tovar J, Tetley L, et al. Characterization of a mitochondrion-like organelle in Cryptosporidium parvum. Parasitology. 2004;129(Pt 1):1–18. doi: 10.1017/s003118200400527x . 15267107

[165] Makiuchi T, Nozaki T. Highly divergent mitochondrion-related organelles in anaerobic parasitic protozoa. Biochimie. 2014;100:3–17. Epub 20131204. doi: 10.1016/j.biochi.2013.11.018 . 24316280

[166] Dagley MJ, Dolezal P, Likic VA, Smid O, Purcell AW, Buchanan SK, et al. The protein import channel in the outer mitosomal membrane of Giardia intestinalis. Mol Biol Evol. 2009;26(9):1941–7. Epub 20090616. doi: 10.1093/molbev/msp117 ; PubMed Central PMCID: PMC2734158. 19531743 PMC2734158

[167] Hjort K, Goldberg AV, Tsaousis AD, Hirt RP, Embley TM. Diversity and reductive evolution of mitochondria among microbial eukaryotes. Philos Trans R Soc Lond Ser B Biol Sci. 2010;365(1541):713–727. doi: 10.1098/rstb.2009.0224 ; PubMed Central PMCID: PMC2817227. 20124340 PMC2817227

[168] Lindmark DG, Müller M. Hydrogenosome, a cytoplasmic organelle of the anaerobic flagellate Tritrichomonas foetus, and its role in pyruvate metabolism. J Biol Chem. 1973;248(22):7724–7728. . 4750424

[169] Bui ET, Bradley PJ, Johnson PJ. A common evolutionary origin for mitochondria and hydrogenosomes. Proc Natl Acad Sci U S A. 1996;93(18):9651–9656. doi: 10.1073/pnas.93.18.9651 ; PubMed Central PMCID: PMC38483. 8790385 PMC38483

[170] Hrdý I, Tachezy J, Müller M. Metabolism of Trichomonad Hydrogenosomes. In: Tachezy J, editor. Hydrogenosomes and Mitosomes: Mitochondria of Anaerobic Eukaryotes. Berlin, Heidelberg: Springer Berlin Heidelberg; 2008. p. 113–45.

[171] Bradley PJ, Lahti CJ, Plümper E, Johnson PJ. Targeting and translocation of proteins into the hydrogenosome of the protist Trichomonas: similarities with mitochondrial protein import. EMBO J. 1997;16(12):3484–3493. doi: 10.1093/emboj/16.12.3484 ; PubMed Central PMCID: PMC1169974. 9218791 PMC1169974

[172] Makki A, Rada P, Žárský V, Kereïche S, Kováčik L, Novotný M, et al. Triplet-pore structure of a highly divergent TOM complex of hydrogenosomes in Trichomonas vaginalis. PLoS Biol. 2019;17(1):e3000098. Epub 20190104. doi: 10.1371/journal.pbio.3000098 ; PubMed Central PMCID: PMC6334971. 30608924 PMC6334971

[173] Rada P, Doležal P, Jedelský PL, Bursac D, Perry AJ, Šedinová M, et al. The core components of organelle biogenesis and membrane transport in the hydrogenosomes of Trichomonas vaginalis. PLoS ONE. 2011;6(9):e24428. Epub 20110915. doi: 10.1371/journal.pone.0024428 ; PubMed Central PMCID: PMC3174187. 21935410 PMC3174187

[174] Vercesi AE, Docampo R, Moreno SN. Energization-dependent Ca2+ accumulation in Trypanosoma brucei bloodstream and procyclic trypomastigotes mitochondria. Mol Biochem Parasitol. 1992;56(2):251–257. doi: 10.1016/0166-6851(92)90174-i . 1484549

[175] Vercesi AE, Docampo R. Ca2+ transport by digitonin-permeabilized Leishmania donovani. Effects of Ca2+, pentamidine and WR-6026 on mitochondrial membrane potential in situ. Biochem J. 1992;284(Pt 2):463–7. doi: 10.1042/bj2840463 ; PubMed Central PMCID: PMC1132661. 1376113 PMC1132661

[176] Basselin M, Robert-Gero M. Alterations in membrane fluidity, lipid metabolism, mitochondrial activity, and lipophosphoglycan expression in pentamidine-resistant Leishmania. Parasitol Res. 1998;84(1):78–83. doi: 10.1007/s004360050361 . 9491432

[177] Uyemura SA, Luo S, Moreno SN, Docampo R. Oxidative phosphorylation, Ca(2+) transport, and fatty acid-induced uncoupling in malaria parasites mitochondria. J Biol Chem. 2000;275(13):9709–9715. doi: 10.1074/jbc.275.13.9709 . 10734123

[178] Uyemura SA, Luo S, Vieira M, Moreno SN, Docampo R. Oxidative phosphorylation and rotenone-insensitive malate- and NADH-quinone oxidoreductases in Plasmodium yoelii yoelii mitochondria in situ. J Biol Chem. 2004;279(1):385–93. Epub 20031015. doi: 10.1074/jbc.M307264200 . 14561763

[179] Tanabe K, Murakami K. Reduction in the mitochondrial membrane potential of Toxoplasma gondii after invasion of host cells. J Cell Sci. 1984;70:73–81. doi: 10.1242/jcs.70.1.73 . 6501438

[180] Vercesi AE, Rodrigues CO, Uyemura SA, Zhong L, Moreno SN. Respiration and oxidative phosphorylation in the apicomplexan parasite Toxoplasma gondii. J Biol Chem. 1998;273(47):31040–31047. doi: 10.1074/jbc.273.47.31040 . 9813002

[181] Reid RA, Moyle J, Mitchell P. Synthesis of Adenosine Triphosphate by a Protonmotive Force in Rat Liver Mitochondria. Nature. 1966;212(5059):257–258. doi: 10.1038/212257a0 5970114

[182] Bausewein T, Mills DJ, Langer JD, Nitschke B, Nussberger S, Kühlbrandt W. Cryo-EM Structure of the TOM Core Complex from Neurospora crassa. Cell. 2017;170(4):693–700.e7. doi: 10.1016/j.cell.2017.07.012 . 28802041

[183] Tucker K, Park E. Cryo-EM structure of the mitochondrial protein-import channel TOM complex at near-atomic resolution. Nat Struct Mol Biol. 2019;26(12):1158–66. Epub 20191118. doi: 10.1038/s41594-019-0339-2 ; PubMed Central PMCID: PMC8439582. 31740857 PMC8439582

[184] Schein SJ, Colombini M, Finkelstein A. Reconstitution in planar lipid bilayers of a voltage-dependent anion-selective channel obtained from paramecium mitochondria. J Membr Biol. 1976;30(2):99–120. doi: 10.1007/BF01869662 . 1011248

[185] Gonçalves RP, Buzhynskyy N, Prima V, Sturgis JN, Scheuring S. Supramolecular assembly of VDAC in native mitochondrial outer membranes. J Mol Biol. 2007;369(2):413–418. doi: 10.1016/j.jmb.2007.03.063 17439818

[186] Najbauer EE, Becker S, Giller K, Zweckstetter M, Lange A, Steinem C, et al. Structure, gating and interactions of the voltage-dependent anion channel. Eur Biophys J. 2021;50(2):159–172. doi: 10.1007/s00249-021-01515-7 33782728 PMC8071794

[187] Zalman LS, Nikaido H, Kagawa Y. Mitochondrial outer membrane contains a protein producing nonspecific diffusion channels. J Biol Chem. 1980;255(5):1771–1774. . 7354054

[188] Ellenrieder L, Dieterle MP, Doan KN, Martensson CU, Floerchinger A, Campo ML, et al. Dual Role of Mitochondrial Porin in Metabolite Transport across the Outer Membrane and Protein Transfer to the Inner Membrane. Mol Cell. 2019;73(5):1056–1065.e7. doi: 10.1016/j.molcel.2018.12.014 WOS:000460545200018. 30738704

[189] Zalk R, Israelson A, Garty ES, Azoulay-Zohar H, Shoshan-Barmatz V. Oligomeric states of the voltage-dependent anion channel and cytochrome c release from mitochondria. Biochem J. 2005;386:73–83. doi: 10.1042/BJ20041356 WOS:000227232900008. 15456403 PMC1134768

[190] Lemasters JJ, Holmuhamedov E. Voltage-dependent anion channel (VDAC) as mitochondrial governator—Thinking outside the box. Biochim Biophys Acta. 2006;1762(2):181–190. doi: 10.1016/j.bbadis.2005.10.006 WOS:000234658900005 16307870

[191] Grevel A, Becker T. Porins as helpers in mitochondrial protein translocation. Biol Chem. 2020;401(6–7):699–708. doi: 10.1515/hsz-2019-0438 WOS:000542162200006. 31967957

[192] Harada T, Sada R, Osugi Y, Matsumoto S, Matsuda T, Hayashi-Nishino M, et al. Palmitoylated CKAP4 regulates mitochondrial functions through an interaction with VDAC2 at ER-mitochondria contact sites. J Cell Sci. 2020;133(21). doi: 10.1242/jcs.249045 33067255

[193] Liu Y, Ma X, Fujioka H, Liu J, Chen S, Zhu X. DJ-1 regulates the integrity and function of ER-mitochondria association through interaction with IP3R3-Grp75-VDAC1. Proc Natl Acad Sci U S A. 2019;116(50):25322–25328. doi: 10.1073/pnas.1906565116 31767755 PMC6911199

[194] Min CK, Yeom DR, Lee KE, Kwon HK, Kang M, Kim YS, et al. Coupling of ryanodine receptor 2 and voltage-dependent anion channel 2 is essential for Ca^2^+ transfer from the sarcoplasmic reticulum to the mitochondria in the heart. Biochem J. 2012;447(3):371–379. doi: 10.1042/bj20120705 . 22867515

[195] Mallo N, Ovciarikova J, Martins-Duarte ES, Baehr SC, Biddau M, Wilde ML, et al. Depletion of a Toxoplasma porin leads to defects in mitochondrial morphology and contacts with the endoplasmic reticulum. J Cell Sci. 2021;134(20). Epub 20211020. doi: 10.1242/jcs.255299 ; PubMed Central PMCID: PMC8572010. 34523684 PMC8572010

[196] Flinner N, Schleiff E, Mirus O. Identification of two voltage-dependent anion channel-like protein sequences conserved in Kinetoplastida. Biol Lett. 2012;8(3):446–9. Epub 20120104. doi: 10.1098/rsbl.2011.1121 ; PubMed Central PMCID: PMC3367756. 22219392 PMC3367756

[197] Pusnik M, Charrière F, Mäser P, Waller RF, Dagley MJ, Lithgow T, et al. The single mitochondrial porin of Trypanosoma brucei is the main metabolite transporter in the outer mitochondrial membrane. Mol Biol Evol. 2009;26(3):671–80. Epub 20081217. doi: 10.1093/molbev/msn288 . 19091722

[198] Bochud-Allemann N, Schneider A. Mitochondrial substrate level phosphorylation is essential for growth of procyclic Trypanosoma brucei. J Biol Chem. 2002;277(36):32849–32854. doi: 10.1074/jbc.M205776200 WOS:000177859000060. 12095995

[199] Dewar CE, Oeljeklaus S, Wenger C, Warscheid B, Schneider A. Characterization of a highly diverged mitochondrial ATP synthase F(o) subunit in Trypanosoma brucei. J Biol Chem. 2022;298(4):101829. Epub 20220312. doi: 10.1016/j.jbc.2022.101829 ; PubMed Central PMCID: PMC9034290. 35293314 PMC9034290

[200] Singha UK, Sharma S, Chaudhuri M. Downregulation of mitochondrial porin inhibits cell growth and alters respiratory phenotype in Trypanosoma brucei. Eukaryot Cell. 2009;8(9):1418–28. Epub 20090717. doi: 10.1128/EC.00132-09 ; PubMed Central PMCID: PMC2747824. 19617393 PMC2747824

[201] De Stefani D, Raffaello A, Teardo E, Szabò I, Rizzuto R. A forty-kilodalton protein of the inner membrane is the mitochondrial calcium uniporter. Nature. 2011;476(7360):336–340. doi: 10.1038/nature10230 21685888 PMC4141877

[202] Pallafacchina G, Zanin S, Rizzuto R. From the Identification to the Dissection of the Physiological Role of the Mitochondrial Calcium Uniporter: An Ongoing Story. Biomol Ther. 2021;11(6). Epub 20210523. doi: 10.3390/biom11060786 ; PubMed Central PMCID: PMC8224590. 34071006 PMC8224590

[203] Docampo R, Vercesi AE. Characteristics of Ca2+ transport by Trypanosoma cruzi mitochondria in situ. Arch Biochem Biophys. 1989;272(1):122–129. doi: 10.1016/0003-9861(89)90202-6 . 2500059

[204] Vercesi AE, Bernardes CF, Hoffmann ME, Gadelha FR, Docampo R. Digitonin permeabilization does not affect mitochondrial function and allows the determination of the mitochondrial membrane potential of Trypanosoma cruzi in situ. J Biol Chem. 1991;266(22):14431–14434. . 1860850

[205] Huang G, Vercesi AE, Docampo R. Essential regulation of cell bioenergetics in Trypanosoma brucei by the mitochondrial calcium uniporter. Nat Commun. 2013;4:2865. doi: 10.1038/ncomms3865 ; PubMed Central PMCID: PMC3868461 24305511 PMC3868461

[206] Huang G, Docampo R. The Mitochondrial Ca(2+) Uniporter Complex (MCUC) of Trypanosoma brucei Is a Hetero-oligomer That Contains Novel Subunits Essential for Ca(2+) Uptake. MBio. 2018;9(5). Epub 20180918. doi: 10.1128/mBio.01700-18 ; PubMed Central PMCID: PMC6143741. 30228243 PMC6143741

[207] Chiurillo MA, Lander N, Bertolini MS, Storey M, Vercesi AE, Docampo R. Different Roles of Mitochondrial Calcium Uniporter Complex Subunits in Growth and Infectivity of Trypanosoma cruzi. MBio. 2017;8(3). Epub 20170509. doi: 10.1128/mBio.00574-17 ; PubMed Central PMCID: PMC5424207. 28487431 PMC5424207

[208] Chiurillo MA, Lander N, Bertolini MS, Vercesi AE, Docampo R. Functional analysis and importance for host cell infection of the Ca(2+)-conducting subunits of the mitochondrial calcium uniporter of Trypanosoma cruzi. Mol Biol Cell. 2019;30(14):1676–90. Epub 20190515. doi: 10.1091/mbc.E19-03-0152 ; PubMed Central PMCID: PMC6727756. 31091170 PMC6727756

[209] Bertolini MS, Chiurillo MA, Lander N, Vercesi AE, Docampo R. MICU1 and MICU2 Play an Essential Role in Mitochondrial Ca(2+) Uptake, Growth, and Infectivity of the Human Pathogen Trypanosoma cruzi. MBio. 2019;10(3). Epub 20190507. doi: 10.1128/mBio.00348-19 ; PubMed Central PMCID: PMC6509184. 31064825 PMC6509184

[210] Mallilankaraman K, Doonan P, Cárdenas C, Chandramoorthy HC, Müller M, Miller R, et al. MICU1 is an essential gatekeeper for MCU-mediated mitochondrial Ca(2+) uptake that regulates cell survival. Cell. 2012;151(3):630–644. doi: 10.1016/j.cell.2012.10.011 ; PubMed Central PMCID: PMC3486697 23101630 PMC3486697

[211] Rizzuto R, Brini M, Murgia M, Pozzan T. Microdomains with high Ca2+ close to IP3-sensitive channels that are sensed by neighboring mitochondria. Science. 1993;262(5134):744–747. doi: 10.1126/science.8235595 . 8235595

[212] Marsault R, Murgia M, Pozzan T, Rizzuto R. Domains of high Ca2+ beneath the plasma membrane of living A7r5 cells. EMBO J. 1997;16(7):1575–81. doi: 10.1093/emboj/16.7.1575 9130702 PMC1169761

[213] Xu H, Guan N, Ren YL, Wei QJ, Tao YH, Yang GS, et al. IP(3)R-Grp75-VDAC1-MCU calcium regulation axis antagonists protect podocytes from apoptosis and decrease proteinuria in an Adriamycin nephropathy rat model. BMC Nephrol. 2018;19(1):140. Epub 20180615. doi: 10.1186/s12882-018-0940-3 ; PubMed Central PMCID: PMC6003198. 29907098 PMC6003198

[214] Lander N, Chiurillo MA, Storey M, Vercesi AE, Docampo R. CRISPR/Cas9-mediated endogenous C-terminal Tagging of Trypanosoma cruzi Genes Reveals the Acidocalcisome Localization of the Inositol 1,4,5-Trisphosphate Receptor. J Biol Chem. 2016;291(49):25505–15. Epub 20161028. doi: 10.1074/jbc.M116.749655 ; PubMed Central PMCID: PMC5207250. 27793988 PMC5207250

[215] Huang G, Bartlett PJ, Thomas AP, Moreno SN, Docampo R. Acidocalcisomes of Trypanosoma brucei have an inositol 1,4,5-trisphosphate receptor that is required for growth and infectivity. Proc Natl Acad Sci U S A. 2013;110(5):1887–92. Epub 20130114. doi: 10.1073/pnas.1216955110 ; PubMed Central PMCID: PMC3562765. 23319604 PMC3562765

[216] Docampo R, Huang G. The IP(3) receptor and Ca(2+) signaling in trypanosomes. Biochim Biophys Acta Mol. Cell Res. 2021;1868(4):118947. Epub 20210106. doi: 10.1016/j.bbamcr.2021.118947 ; PubMed Central PMCID: PMC7882029. 33421534 PMC7882029

[217] Docampo R, Vercesi AE, Huang G, Lander N, Chiurillo MA, Bertolini M. Mitochondrial Ca(2+) homeostasis in trypanosomes. Int Rev Cell Mol Biol. 2021;362:261–289. doi: 10.1016/bs.ircmb.2021.01.002 34253297 PMC10424509

[218] Benaim G, Paniz-Mondolfi AE, Sordillo EM, Martinez-Sotillo N. Disruption of Intracellular Calcium Homeostasis as a Therapeutic Target Against Trypanosoma cruzi. Front Cell Infect Microbiol. 2020;10:46. doi: 10.3389/fcimb.2020.00046 32133302 PMC7040492

[219] Alleva LM, Kirk K. Calcium regulation in the intraerythrocytic malaria parasite Plasmodium falciparum. Mol Biochem Parasitol. 2001;117(2):121–128. doi: 10.1016/s0166-6851(01)00338-3 . 11606221

[220] Rotmann A, Sanchez C, Guiguemde A, Rohrbach P, Dave A, Bakouh N, et al. PfCHA is a mitochondrial divalent cation/H+ antiporter in Plasmodium falciparum. Mol Microbiol. 2010;76(6):1591–606. Epub 20100504. doi: 10.1111/j.1365-2958.2010.07187.x . 20487273

[221] Bick AG, Calvo SE, Mootha VK. Evolutionary diversity of the mitochondrial calcium uniporter. Science. 2012;336(6083):886. doi: 10.1126/science.1214977 ; PubMed Central PMCID: PMC3518847. 22605770 PMC3518847

[222] Biagini GA, Bray PG, Spiller DG, White MR, Ward SA. The digestive food vacuole of the malaria parasite is a dynamic intracellular Ca2+ store. J Biol Chem. 2003;278(30):27910–5. Epub 20030508. doi: 10.1074/jbc.M304193200 . 12740366

[223] Miranda K, Pace DA, Cintron R, Rodrigues JC, Fang J, Smith A, et al. Characterization of a novel organelle in Toxoplasma gondii with similar composition and function to the plant vacuole. Mol Microbiol. 2010;76(6):1358–75. Epub 20100414. doi: 10.1111/j.1365-2958.2010.07165.x ; PubMed Central PMCID: PMC2907454. 20398214 PMC2907454

[224] Patel S, Docampo R. Acidic calcium stores open for business: expanding the potential for intracellular Ca2+ signaling. Trends Cell Biol. 2010;20(5):277–86. Epub 20100318. doi: 10.1016/j.tcb.2010.02.003 ; PubMed Central PMCID: PMC2862797. 20303271 PMC2862797

[225] Docampo R, Scott DA, Vercesi AE, Moreno SN. Intracellular Ca2+ storage in acidocalcisomes of Trypanosoma cruzi. Biochem J. 1995;310(Pt 3):1005–12. doi: 10.1042/bj3101005 ; PubMed Central PMCID: PMC1135995. 7575396 PMC1135995

[226] Vercesi AE, Moreno SN, Docampo R. Ca2+/H+ exchange in acidic vacuoles of Trypanosoma brucei. Biochem J. 1994;304(Pt 1):227–233. doi: 10.1042/bj3040227 ; PubMed Central PMCID: PMC1137476. 7998937 PMC1137476

[227] Moreno SN, Zhong L. Acidocalcisomes in Toxoplasma gondii tachyzoites. Biochem J. 1996;313(Pt 2):655–659. doi: 10.1042/bj3130655 ; PubMed Central PMCID: PMC1216957. 8573106 PMC1216957

[228] Ruiz FA, Luo S, Moreno SN, Docampo R. Polyphosphate content and fine structure of acidocalcisomes of Plasmodium falciparum. Microsc Microanal. 2004;10(5):563–567. doi: 10.1017/S1431927604040875 . 15525430

[229] Soares Medeiros LC, Gomes F, Maciel LR, Seabra SH, Docampo R, Moreno S, et al. Volutin granules of Eimeria parasites are acidic compartments and have physiological and structural characteristics similar to acidocalcisomes. J Eukaryot Microbiol. 2011;58(5):416–23. Epub 20110623. doi: 10.1111/j.1550-7408.2011.00565.x ; PubMed Central PMCID: PMC3443593. 21699625 PMC3443593

[230] Docampo R, Moreno SN. The acidocalcisome. Mol Biochem Parasitol. 2001;114(2):151–159. doi: 10.1016/s0166-6851(01)00246-8 . 11378195

[231] Seufferheld M, Vieira MC, Ruiz FA, Rodrigues CO, Moreno SN, Docampo R. Identification of organelles in bacteria similar to acidocalcisomes of unicellular eukaryotes. J Biol Chem. 2003;278(32):29971–8. Epub 20030603. doi: 10.1074/jbc.M304548200 . 12783865

[232] Marchesini N, Ruiz FA, Vieira M, Docampo R. Acidocalcisomes are functionally linked to the contractile vacuole of Dictyostelium discoideum. J Biol Chem. 2002;277(10):8146–53. Epub 20011217. doi: 10.1074/jbc.M111130200 . 11748243

[233] Ruiz FA, Marchesini N, Seufferheld M, Govindjee, Docampo R. The polyphosphate bodies of Chlamydomonas reinhardtii possess a proton-pumping pyrophosphatase and are similar to acidocalcisomes. J Biol Chem. 2001;276(49):46196–203. Epub 20010928. doi: 10.1074/jbc.M105268200 . 11579086

[234] Luo S, Rohloff P, Cox J, Uyemura SA, Docampo R. Trypanosoma brucei plasma membrane-type Ca(2+)-ATPase 1 (TbPMC1) and 2 (TbPMC2) genes encode functional Ca(2+)-ATPases localized to the acidocalcisomes and plasma membrane, and essential for Ca(2+) homeostasis and growth. J Biol Chem. 2004;279(14):14427–39. Epub 20040114. doi: 10.1074/jbc.M309978200 . 14724285

[235] Luo S, Ruiz FA, Moreno SN. The acidocalcisome Ca2+-ATPase (TgA1) of Toxoplasma gondii is required for polyphosphate storage, intracellular calcium homeostasis and virulence. Mol Microbiol. 2005;55(4):1034–1045. doi: 10.1111/j.1365-2958.2004.04464.x . 15686552

[236] Lu HG, Zhong L, de Souza W, Benchimol M, Moreno S, Docampo R. Ca2+ content and expression of an acidocalcisomal calcium pump are elevated in intracellular forms of Trypanosoma cruzi. Mol Cell Biol. 1998;18(4):2309–2323. doi: 10.1128/MCB.18.4.2309 ; PubMed Central PMCID: PMC121484. 9528801 PMC121484

[237] Potapenko E, Negrão NW, Huang G, Docampo R. The acidocalcisome inositol-1,4,5-trisphosphate receptor of Trypanosoma brucei is stimulated by luminal polyphosphate hydrolysis products. J Biol Chem. 2019;294(27):10628–37. Epub 20190528. doi: 10.1074/jbc.RA119.007906 ; PubMed Central PMCID: PMC6615701. 31138655 PMC6615701

[238] Lemercier G, Espiau B, Ruiz FA, Vieira M, Luo S, Baltz T, et al. A pyrophosphatase regulating polyphosphate metabolism in acidocalcisomes is essential for Trypanosoma brucei virulence in mice. J Biol Chem. 2004;279(5):3420–5. Epub 20031113. doi: 10.1074/jbc.M309974200 . 14615483

[239] Rohloff P, Docampo R. Ammonium production during hypo-osmotic stress leads to alkalinization of acidocalcisomes and cytosolic acidification in Trypanosoma cruzi. Mol Biochem Parasitol. 2006;150(2):249–55. Epub 20060915. doi: 10.1016/j.molbiopara.2006.08.010 . 17005261

[240] Docampo R, Huang G. New insights into the role of acidocalcisomes in trypanosomatids. J Eukaryot Microbiol. 2022:e12899. Epub 20220221. doi: 10.1111/jeu.12899 . 35191563 PMC10350294

[241] Alves E, Bartlett PJ, Garcia CR, Thomas AP. Melatonin and IP3-induced Ca2+ release from intracellular stores in the malaria parasite Plasmodium falciparum within infected red blood cells. J Biol Chem. 2011;286(7):5905–12. Epub 20101213. doi: 10.1074/jbc.M110.188474 ; PubMed Central PMCID: PMC3037703. 21149448 PMC3037703

[242] Chini EN, Nagamune K, Wetzel DM, Sibley LD. Evidence that the cADPR signalling pathway controls calcium-mediated microneme secretion in Toxoplasma gondii. Biochem J. 2005;389(Pt 2):269–277. doi: 10.1042/BJ20041971 ; PubMed Central PMCID: PMC1175103. 15773818 PMC1175103

[243] Fang J, Marchesini N, Moreno SN. A Toxoplasma gondii phosphoinositide phospholipase C (TgPI-PLC) with high affinity for phosphatidylinositol. Biochem J. 2006;394(Pt 2):417–425. doi: 10.1042/BJ20051393 ; PubMed Central PMCID: PMC1408672. 16288600 PMC1408672

[244] Bullen HE, Jia Y, Yamaryo-Botté Y, Bisio H, Zhang O, Jemelin NK, et al. Phosphatidic Acid-Mediated Signaling Regulates Microneme Secretion in Toxoplasma. Cell Host Microbe. 2016;19(3):349–360. doi: 10.1016/j.chom.2016.02.006 . 26962945

[245] Garcia CRS, Alves E, Pereira PHS, Bartlett PJ, Thomas AP, Mikoshiba K, et al. InsP3 Signaling in Apicomplexan Parasites. Curr Top Med Chem. 2017;17(19):2158–2165. doi: 10.2174/1568026617666170130121042 ; PubMed Central PMCID: PMC5490149. 28137231 PMC5490149

[246] Steinmann ME, Schmidt RS, Bütikofer P, Mäser P, Sigel E. TbIRK is a signature sequence free potassium channel from Trypanosoma brucei locating to acidocalcisomes. Sci Rep. 2017;7(1):656. Epub 20170406. doi: 10.1038/s41598-017-00752-1 ; PubMed Central PMCID: PMC5429665. 28386071 PMC5429665

[247] Jimenez V, Miranda K, Augusto I. The old and the new about the contractile vacuole of Trypanosoma cruzi. J Eukaryot Microbiol. 2022:e12939. Epub 20220802. doi: 10.1111/jeu.12939 . 35916682 PMC11178379

[248] Prole DL, Taylor CW. Identification and analysis of putative homologues of mechanosensitive channels in pathogenic protozoa. PLoS ONE. 2013;8(6):e66068. Epub 20130613. doi: 10.1371/journal.pone.0066068 ; PubMed Central PMCID: PMC3681921. 23785469 PMC3681921

[249] Wang X, Zhang X, Dong XP, Samie M, Li X, Cheng X, et al. TPC proteins are phosphoinositide- activated sodium-selective ion channels in endosomes and lysosomes. Cell. 2012;151(2):372–383. doi: 10.1016/j.cell.2012.08.036 ; PubMed Central PMCID: PMC3475186. 23063126 PMC3475186

[250] Brohawn SG, Su Z, MacKinnon R. Mechanosensitivity is mediated directly by the lipid membrane in TRAAK and TREK1 K+ channels. Proc Natl Acad Sci U S A. 2014;111(9):3614–9. Epub 20140218. doi: 10.1073/pnas.1320768111 ; PubMed Central PMCID: PMC3948252. 24550493 PMC3948252

[251] Chen CC, Krogsaeter E, Grimm C. Two-pore and TRP cation channels in endolysosomal osmo-/mechanosensation and volume regulation. Biochim Biophys Acta Mol. Cell Res. 2021;1868(2):118921. Epub 20201203. doi: 10.1016/j.bbamcr.2020.118921 . 33279607

[252] Martins-Duarte É S, Sheiner L, Reiff SB, de Souza W, Striepen B. Replication and partitioning of the apicoplast genome of Toxoplasma gondii is linked to the cell cycle and requires DNA polymerase and gyrase. Int J Parasitol. 2021;51(6):493–504. Epub 20210211. doi: 10.1016/j.ijpara.2020.11.004 ; PubMed Central PMCID: PMC8113025. 33581138 PMC8113025

[253] Ramakrishnan S, Docampo MD, Macrae JI, Pujol FM, Brooks CF, van Dooren GG, et al. Apicoplast and endoplasmic reticulum cooperate in fatty acid biosynthesis in apicomplexan parasite Toxoplasma gondii. J Biol Chem. 2012;287(7):4957–71. Epub 20111216. doi: 10.1074/jbc.M111.310144 ; PubMed Central PMCID: PMC3281623. 22179608 PMC3281623

[254] Striepen B. The apicoplast: a red alga in human parasites. Essays Biochem. 2011;51:111–125. doi: 10.1042/bse0510111 . 22023445

[255] Reiff SB, Vaishnava S, Striepen B. The HU protein is important for apicoplast genome maintenance and inheritance in Toxoplasma gondii. Eukaryot Cell. 2012;11(7):905–15. Epub 20120518. doi: 10.1128/EC.00029-12 ; PubMed Central PMCID: PMC3416497. 22611021 PMC3416497

[256] Ginsburg H, Krugliak M, Eidelman O, Cabantchik ZI. New permeability pathways induced in membranes of Plasmodium falciparum infected erythrocytes. Mol Biochem Parasitol. 1983;8(2):177–190. doi: 10.1016/0166-6851(83)90008-7 . 6348537

[257] Ho CM, Beck JR, Lai M, Cui Y, Goldberg DE, Egea PF, et al. Malaria parasite translocon structure and mechanism of effector export. Nature. 2018;561(7721):70–5. Epub 20180827. doi: 10.1038/s41586-018-0469-4 ; PubMed Central PMCID: PMC6555636. 30150771 PMC6555636

[258] Beck JR, Muralidharan V, Oksman A, Goldberg DE. PTEX component HSP101 mediates export of diverse malaria effectors into host erythrocytes. Nature. 2014;511(7511):592–5. Epub 20140716. doi: 10.1038/nature13574 ; PubMed Central PMCID: PMC4130291. 25043010 PMC4130291

[259] de Koning-Ward TF, Gilson PR, Boddey JA, Rug M, Smith BJ, Papenfuss AT, et al. A newly discovered protein export machine in malaria parasites. Nature. 2009;459(7249):945–949. doi: 10.1038/nature08104 ; PubMed Central PMCID: PMC2725363. 19536257 PMC2725363

[260] Jonsdottir TK, Gabriela M, Crabb BS, T FdK-W, Gilson PR. Defining the Essential Exportome of the Malaria Parasite. Trends Parasitol. 2021;37(7):664–75. Epub 20210510. doi: 10.1016/j.pt.2021.04.009 . 33985912

[261] Staines HM, Ellory JC, Kirk K. Perturbation of the pump-leak balance for Na(+) and K(+) in malaria-infected erythrocytes. Am J Phys Cell Phys. 2001;280(6):C1576–C1587. doi: 10.1152/ajpcell.2001.280.6.C1576 . 11350753

[262] Staines HM, Rae C, Kirk K. Increased permeability of the malaria-infected erythrocyte to organic cations. Biochim Biophys Acta. 2000;1463(1):88–98. doi: 10.1016/s0005-2736(99)00187-x . 10631297

[263] Duranton C, Huber SM, Tanneur V, Brand VB, Akkaya C, Shumilina EV, et al. Organic osmolyte permeabilities of the malaria-induced anion conductances in human erythrocytes. J Gen Physiol. 2004;123(4):417–426. doi: 10.1085/jgp.200308919 ; PubMed Central PMCID: PMC2217455. 15051807 PMC2217455

[264] Desai SA, Bezrukov SM, Zimmerberg J. A voltage-dependent channel involved in nutrient uptake by red blood cells infected with the malaria parasite. Nature. 2000;406(6799):1001–1005. doi: 10.1038/35023000 . 10984055

[265] Nguitragool W, Bokhari AA, Pillai AD, Rayavara K, Sharma P, Turpin B, et al. Malaria parasite clag3 genes determine channel-mediated nutrient uptake by infected red blood cells. Cell. 2011;145(5):665–677. doi: 10.1016/j.cell.2011.05.002 ; PubMed Central PMCID: PMC3105333. 21620134 PMC3105333

[266] Ahmad M, Manzella-Lapeira J, Saggu G, Ito D, Brzostowski JA, Desai SA. Live-Cell FRET Reveals that Malaria Nutrient Channel Proteins CLAG3 and RhopH2 Remain Associated throughout Their Tortuous Trafficking. MBio. 2020;11(5). Epub 20200908. doi: 10.1128/mBio.01354-20 ; PubMed Central PMCID: PMC7482060. 32900800 PMC7482060

[267] Schureck MA, Darling JE, Merk A, Shao J, Daggupati G, Srinivasan P, et al. Malaria parasites use a soluble RhopH complex for erythrocyte invasion and an integral form for nutrient uptake. elife. 2021;10. Epub 20210104. doi: 10.7554/eLife.65282 ; PubMed Central PMCID: PMC7840181. 33393463 PMC7840181

[268] Pasternak M, Verhoef JMJ, Wong W, Triglia T, Mlodzianoski MJ, Geoghegan N, et al. RhopH2 and RhopH3 export enables assembly of the RhopH complex on P. falciparum-infected erythrocyte membranes. Commun Biol. 2022;5(1):333. Epub 20220407. doi: 10.1038/s42003-022-03290-3 ; PubMed Central PMCID: PMC8989874. 35393572 PMC8989874

[269] Kirk K, Horner HA, Elford BC, Ellory JC, Newbold CI. Transport of diverse substrates into malaria-infected erythrocytes via a pathway showing functional characteristics of a chloride channel. J Biol Chem. 1994;269(5):3339–3347. . 8106373

[270] Gupta A, Balabaskaran-Nina P, Nguitragool W, Saggu GS, Schureck MA, Desai SA. CLAG3 Self-Associates in Malaria Parasites and Quantitatively Determines Nutrient Uptake Channels at the Host Membrane. MBio. 2018;9(3). Epub 20180508. doi: 10.1128/mBio.02293-17 ; PubMed Central PMCID: PMC5941077. 29739907 PMC5941077

[271] Gupta A, Bokhari AAB, Pillai AD, Crater AK, Gezelle J, Saggu G, et al. Complex nutrient channel phenotypes despite Mendelian inheritance in a Plasmodium falciparum genetic cross. PLoS Pathog. 2020;16(2):e1008363. Epub 20200218. doi: 10.1371/journal.ppat.1008363 ; PubMed Central PMCID: PMC7048409. 32069335 PMC7048409

[272] Ginsburg H. The permeability properties of the parasite cell membrane. Novartis Found Symp. 1999;226:99–108; discussion 108–13. doi: 10.1002/9780470515730.ch8 . 10645541

[273] Saliba KJ, Martin RE, Bröer A, Henry RI, McCarthy CS, Downie MJ, et al. Sodium-dependent uptake of inorganic phosphate by the intracellular malaria parasite. Nature. 2006;443(7111):582–5. Epub 20060927. doi: 10.1038/nature05149 . 17006451

[274] Cohn JV, Alkhalil A, Wagner MA, Rajapandi T, Desai SA. Extracellular lysines on the plasmodial surface anion channel involved in Na+ exclusion. Mol Biochem Parasitol. 2003;132(1):27–34. doi: 10.1016/j.molbiopara.2003.08.001 . 14563534

[275] Ginsburg H, Kutner S, Krugliak M, Cabantchik ZI. Characterization of permeation pathways appearing in the host membrane of Plasmodium falciparum infected red blood cells. Mol Biochem Parasitol. 1985;14(3):313–322. doi: 10.1016/0166-6851(85)90059-3 . 3887158

[276] Saliba KJ, Horner HA, Kirk K. Transport and metabolism of the essential vitamin pantothenic acid in human erythrocytes infected with the malaria parasite Plasmodium falciparum. J Biol Chem. 1998;273(17):10190–10195. doi: 10.1074/jbc.273.17.10190 . 9553068

[277] Upston JM, Gero AM. Parasite-induced permeation of nucleosides in Plasmodium falciparum malaria. Biochim Biophys Acta. 1995;1236(2):249–258. doi: 10.1016/0005-2736(95)00055-8 . 7794964

[278] Staines HM, Alkhalil A, Allen RJ, De Jonge HR, Derbyshire E, Egée S, et al. Electrophysiological studies of malaria parasite-infected erythrocytes: current status. Int J Parasitol. 2007;37(5):475–82. Epub 20070109. doi: 10.1016/j.ijpara.2006.12.013 ; PubMed Central PMCID: PMC2746352. 17292372 PMC2746352

[279] Huber SM, Duranton C, Lang F. Patch-clamp analysis of the "new permeability pathways" in malaria-infected erythrocytes. Int Rev Cytol. 2005;246:59–134. doi: 10.1016/S0074-7696(05)46003-9 . 16164967

[280] Huber SM, Duranton C, Henke G, Van De Sand C, Heussler V, Shumilina E, et al. Plasmodium induces swelling-activated ClC-2 anion channels in the host erythrocyte. J Biol Chem. 2004;279(40):41444–52. Epub 20040721. doi: 10.1074/jbc.M407618200 . 15272009

[281] Bouyer G, Cueff A, Egée S, Kmiecik J, Maksimova Y, Glogowska E, et al. Erythrocyte peripheral type benzodiazepine receptor/voltage-dependent anion channels are upregulated by Plasmodium falciparum. Blood. 2011;118(8):2305–12. Epub 20110727. doi: 10.1182/blood-2011-01-329300 . 21795748

[282] Jonsdottir TK, Counihan NA, Modak JK, Kouskousis B, Sanders PR, Gabriela M, et al. Characterisation of complexes formed by parasite proteins exported into the host cell compartment of Plasmodium falciparum infected red blood cells. Cell Microbiol. 2021;23(8):e13332. Epub 20210503. doi: 10.1111/cmi.13332 ; PubMed Central PMCID: PMC8365696. 33774908 PMC8365696

[283] Garten M, Nasamu AS, Niles JC, Zimmerberg J, Goldberg DE, Beck JR. EXP2 is a nutrient-permeable channel in the vacuolar membrane of Plasmodium and is essential for protein export via PTEX. Nat Microbiol. 2018;3(10):1090–8. Epub 20180827. doi: 10.1038/s41564-018-0222-7 ; PubMed Central PMCID: PMC6158082. 30150733 PMC6158082

[284] Desai SA, Rosenberg RL. Pore size of the malaria parasite’s nutrient channel. Proc Natl Acad Sci U S A. 1997;94(5):2045–2049. doi: 10.1073/pnas.94.5.2045 ; PubMed Central PMCID: PMC20040. 9050902 PMC20040

[285] Garten M, Beck JR. Structured to conquer: transport across the Plasmodium parasitophorous vacuole. Curr Opin Microbiol. 2021;63:181–8. Epub 20210807. doi: 10.1016/j.mib.2021.07.010 ; PubMed Central PMCID: PMC8463430. 34375857 PMC8463430

[286] Mesén-Ramírez P, Bergmann B, Tran TT, Garten M, Stäcker J, Naranjo-Prado I, et al. EXP1 is critical for nutrient uptake across the parasitophorous vacuole membrane of malaria parasites. PLoS Biol. 2019;17(9):e3000473. Epub 20190930. doi: 10.1371/journal.pbio.3000473 ; PubMed Central PMCID: PMC6786648. 31568532 PMC6786648

[287] Gold Daniel A, Kaplan Aaron D, Lis A, Bett Glenna CL, Rosowski Emily E, Cirelli Kimberly M, et al. The Toxoplasma Dense Granule Proteins GRA17 and GRA23 Mediate the Movement of Small Molecules between the Host and the Parasitophorous Vacuole. Cell Host Microbe. 2015;17(5):642–52. doi: 10.1016/j.chom.2015.04.003 25974303 PMC4435723

[288] Paredes-Santos T, Wang Y, Waldman B, Lourido S, Saeij JP. The GRA17 Parasitophorous Vacuole Membrane Permeability Pore Contributes to Bradyzoite Viability. Front Cell Infect Microbiol. 2019;9:321. Epub 20190912. doi: 10.3389/fcimb.2019.00321 ; PubMed Central PMCID: PMC6751312. 31572690 PMC6751312

[289] Li TT, Wang JL, Liang QL, Sun LX, Zhang HS, Zhang ZW, et al. Effect of deletion of gra17 and gra23 genes on the growth, virulence, and immunogenicity of type II Toxoplasma gondii. Parasitol Res. 2020;119(9):2907–16. Epub 20200720. doi: 10.1007/s00436-020-06815-z . 32686022

[290] Castro R, Scott K, Jordan T, Evans B, Craig J, Peters EL, et al. The ultrastructure of the parasitophorous vacuole formed by Leishmania major. J Parasitol. 2006;92(6):1162–1170. doi: 10.1645/GE-841R.1 . 17304790

[291] Sarkar A, Khan YA, Laranjeira-Silva MF, Andrews NW, Mittra B. Quantification of Intracellular Growth Inside Macrophages is a Fast and Reliable Method for Assessing the Virulence of Leishmania Parasites. J Vis Exp. 2018;(133). Epub 20180316. doi: 10.3791/57486 ; PubMed Central PMCID: PMC5931781. 29608175 PMC5931781

[292] Ley V, Robbins ES, Nussenzweig V, Andrews NW. The exit of Trypanosoma cruzi from the phagosome is inhibited by raising the pH of acidic compartments. J Exp Med. 1990;171(2):401–413. doi: 10.1084/jem.171.2.401 2406362 PMC2187728

[293] Henriques C, de Souza W. Redistribution of plasma-membrane surface molecules during formation of the Leishmania amazonensis-containing parasitophorous vacuole. Parasitol Res. 2000;86(3):215–225. doi: 10.1007/s004360050034 . 10726992

[294] Naderer T, Heng J, McConville MJ. Evidence that intracellular stages of Leishmania major utilize amino sugars as a major carbon source. PLoS Pathog. 2010;6(12):e1001245. Epub 20101223. doi: 10.1371/journal.ppat.1001245 ; PubMed Central PMCID: PMC3009595. 21203480 PMC3009595

[295] Naderer T, Heng J, Saunders EC, Kloehn J, Rupasinghe TW, Brown TJ, et al. Intracellular Survival of Leishmania major Depends on Uptake and Degradation of Extracellular Matrix Glycosaminoglycans by Macrophages. PLoS Pathog. 2015;11(9):e1005136. Epub 20150903. doi: 10.1371/journal.ppat.1005136 ; PubMed Central PMCID: PMC4559419. 26334531 PMC4559419

[296] Saunders EC, Naderer T, Chambers J, Landfear SM, McConville MJ. Leishmania mexicana can utilize amino acids as major carbon sources in macrophages but not in animal models. Mol Microbiol. 2018;108(2):143–58. Epub 20180302. doi: 10.1111/mmi.13923 ; PubMed Central PMCID: PMC7428322. 29411460 PMC7428322

[297] Živanović V, Semini G, Laue M, Drescher D, Aebischer T, Kneipp J. Chemical Mapping of Leishmania Infection in Live Cells by SERS Microscopy. Anal Chem. 2018;90(13):8154–61. Epub 20180619. doi: 10.1021/acs.analchem.8b01451 . 29870219

[298] Young J, Kima PE. The Leishmania Parasitophorous Vacuole Membrane at the Parasite-Host Interface. Yale J Biol Med. 2019;92(3):511–521. Epub 20190920. doi: 10.1371/journal.pone.0035671 ; PubMed Central PMCID: PMC6747952. 31543712 PMC6747952

[299] Tardieux I, Webster P, Ravesloot J, Boron W, Lunn JA, Heuser JE, et al. Lysosome recruitment and fusion are early events required for trypanosome invasion of mammalian cells. Cell. 1992;71(7):1117–1130. doi: 10.1016/s0092-8674(05)80061-3 . 1473148

[300] Woolsey AM, Sunwoo L, Petersen CA, Brachmann SM, Cantley LC, Burleigh BA. Novel PI 3-kinase-dependent mechanisms of trypanosome invasion and vacuole maturation. J Cell Sci. 2003;116(Pt 17):3611–22. Epub 20030722. doi: 10.1242/jcs.00666 . 12876217

[301] Andrews NW, Abrams CK, Slatin SL, Griffiths G A T. cruzi-secreted protein immunologically related to the complement component C9: evidence for membrane pore-forming activity at low pH. Cell. 1990;61(7):1277–1287. doi: 10.1016/0092-8674(90)90692-8 . 2194668

[302] Zdrazil B, Richter L, Brown N, Guha R. Moving targets in drug discovery. Sci Rep. 2020;10(1):20213. doi: 10.1038/s41598-020-77033-x 33214619 PMC7677539

[303] Blount P, Iscla I. Life with Bacterial Mechanosensitive Channels, from Discovery to Physiology to Pharmacological Target. Microbiol Mol Biol Rev. 2020;84(1). Epub 20200115. doi: 10.1128/MMBR.00055-19 ; PubMed Central PMCID: PMC7167205. 31941768 PMC7167205

[304] Machado D, Pires D, Perdigão J, Couto I, Portugal I, Martins M, et al. Ion Channel Blockers as Antimicrobial Agents, Efflux Inhibitors, and Enhancers of Macrophage Killing Activity against Drug Resistant Mycobacterium tuberculosis. PLoS ONE. 2016;11(2):e0149326. Epub 20160226. doi: 10.1371/journal.pone.0149326 ; PubMed Central PMCID: PMC4769142. 26919135 PMC4769142

[305] Charlton FW, Pearson HM, Hover S, Lippiat JD, Fontana J, Barr JN, et al. Ion Channels as Therapeutic Targets for Viral Infections: Further Discoveries and Future Perspectives. Viruses. 2020;12(8). Epub 20200803. doi: 10.3390/v12080844 ; PubMed Central PMCID: PMC7472218. 32756358 PMC7472218

[306] Meier A, Erler H, Beitz E. Targeting Channels and Transporters in Protozoan Parasite Infections. Front Chem. 2018;6:88. Epub 20180327. doi: 10.3389/fchem.2018.00088 ; PubMed Central PMCID: PMC5881087. 29637069 PMC5881087

[307] Michiels CF, Van Hove CE, Martinet W, De Meyer GR, Fransen P. L-type Ca2+ channel blockers inhibit the window contraction of mouse aorta segments with high affinity. Eur J Pharmacol. 2014;738:170–8. Epub 20140602. doi: 10.1016/j.ejphar.2014.05.036 . 24886884

[308] Silke B, Goldhammer E, Sharma SK, Verma SP, Taylor SH. An exercise hemodynamic comparison of verapamil, diltiazem, and amlodipine in coronary artery disease. Cardiovasc Drugs Ther. 1990;4(2):457–463. doi: 10.1007/BF01857754 . 2149514

[309] Mantegazza M, Curia G, Biagini G, Ragsdale DS, Avoli M. Voltage-gated sodium channels as therapeutic targets in epilepsy and other neurological disorders. Lancet Neurol. 2010;9(4):413–424. doi: 10.1016/S1474-4422(10)70059-4 . 20298965

[310] Patel R, Dickenson AH. Mechanisms of the gabapentinoids and α 2 δ-1 calcium channel subunit in neuropathic pain. Pharmacol Res Perspect. 2016;4(2):e00205. Epub 20160227. doi: 10.1002/prp2.205 ; PubMed Central PMCID: PMC4804325. 27069626 PMC4804325

[311] Wisedchaisri G, Gamal El-Din TM. Druggability of Voltage-Gated Sodium Channels-Exploring Old and New Drug Receptor Sites. Front Pharmacol. 2022;13:858348. Epub 20220317. doi: 10.3389/fphar.2022.858348 ; PubMed Central PMCID: PMC8968173. 35370700 PMC8968173

[312] Carazo-Arias E, Nguyen PT, Kass M, Jee HJ, Nautiyal KM, Magalong V, et al. Contribution of the Opioid System to the Antidepressant Effects of Fluoxetine. Biol Psychiatry. 2022. Epub 20220614. doi: 10.1016/j.biopsych.2022.05.030 . 35977861 PMC10426813

[313] Fluyau D, Mitra P, Jain A, Kailasam VK, Pierre CG. Selective serotonin reuptake inhibitors in the treatment of depression, anxiety, and post-traumatic stress disorder in substance use disorders: a Bayesian meta-analysis. Eur J Clin Pharmacol. 2022;78(6):931–42. Epub 20220305. doi: 10.1007/s00228-022-03303-4 . 35246699

[314] Shi HJ, Wu DL, Chen R, Li N, Zhu LJ. Requirement of hippocampal DG nNOS-CAPON dissociation for the anxiolytic and antidepressant effects of fluoxetine. Theranostics. 2022;12(8):3656–75. Epub 20220501. doi: 10.7150/thno.70370 ; PubMed Central PMCID: PMC9131266. 35664081 PMC9131266

[315] Park SK, Gunaratne GS, Chulkov EG, Moehring F, McCusker P, Dosa PI, et al. The anthelmintic drug praziquantel activates a schistosome transient receptor potential channel. J Biol Chem. 2019;294(49):18873–80. Epub 20191025. doi: 10.1074/jbc.AC119.011093 ; PubMed Central PMCID: PMC6901322. 31653697 PMC6901322

[316] Cully DF, Vassilatis DK, Liu KK, Paress PS, Van der Ploeg LH, Schaeffer JM, et al. Cloning of an avermectin-sensitive glutamate-gated chloride channel from Caenorhabditis elegans. Nature. 1994;371(6499):707–711. doi: 10.1038/371707a0 . 7935817

[317] Laing R, Gillan V, Devaney E. Ivermectin—Old Drug, New Tricks? Trends Parasitol. 2017;33(6):463–72. Epub 20170309. doi: 10.1016/j.pt.2017.02.004 ; PubMed Central PMCID: PMC5446326. 28285851 PMC5446326

[318] Abongwa M, Martin RJ, Robertson AP. A BRIEF REVIEW ON THE MODE OF ACTION OF ANTINEMATODAL DRUGS. Acta Vet (Beograd). 2017;67(2):137–52. Epub 20170626. doi: 10.1515/acve-2017-0013 ; PubMed Central PMCID: PMC5798647. 29416226 PMC5798647

[319] Choudhary S, Kashyap SS, Martin RJ, Robertson AP. Advances in our understanding of nematode ion channels as potential anthelmintic targets. Int J Parasitol Drugs Drug Resist. 2022;18:52–86. Epub 20211225. doi: 10.1016/j.ijpddr.2021.12.001 ; PubMed Central PMCID: PMC8841521. 35149380 PMC8841521

[320] Eckstein-Ludwig U, Webb RJ, Van Goethem ID, East JM, Lee AG, Kimura M, et al. Artemisinins target the SERCA of Plasmodium falciparum. Nature. 2003;424(6951):957–961. doi: 10.1038/nature01813 . 12931192

[321] Krishna S, Pulcini S, Moore CM, Teo BH, Staines HM. Pumped up: reflections on PfATP6 as the target for artemisinins. Trends Pharmacol Sci. 2014;35(1):4–11. Epub 20131121. doi: 10.1016/j.tips.2013.10.007 . 24268763

[322] O’Neill PM, Barton VE, Ward SA. The Molecular Mechanism of Action of Artemisinin—The Debate Continues. Molecules. 2010;15(3):1705–1721. doi: 10.3390/molecules15031705 20336009 PMC6257357

[323] Painter HJ, Morrisey JM, Vaidya AB. Mitochondrial electron transport inhibition and viability of intraerythrocytic Plasmodium falciparum. Antimicrob Agents Chemother. 2010;54(12):5281–7. Epub 20100920. doi: 10.1128/AAC.00937-10 ; PubMed Central PMCID: PMC2981233. 20855748 PMC2981233

[324] Srivastava IK, Rottenberg H, Vaidya AB. Atovaquone, a broad spectrum antiparasitic drug, collapses mitochondrial membrane potential in a malarial parasite. J Biol Chem. 1997;272(7):3961–3966. doi: 10.1074/jbc.272.7.3961 . 9020100

[325] Benaim B, Garcia CR. Targeting calcium homeostasis as the therapy of Chagas’ disease and leishmaniasis—a review. Trop Biomed. 2011;28(3):471–481. . 22433874

[326] Gupta Y, Goicoechea S, Pearce CM, Mathur R, Romero JG, Kwofie SK, et al. The emerging paradigm of calcium homeostasis as a new therapeutic target for protozoan parasites. Med Res Rev. 2022;42(1):56–82. Epub 20210413. doi: 10.1002/med.21804 . 33851452

[327] Pan JQ, Baez-Nieto D, Allen A, Wang HR, Cottrell JR. Developing High-Throughput Assays to Analyze and Screen Electrophysiological Phenotypes. Methods Mol Biol. 2018;1787:235–252. doi: 10.1007/978-1-4939-7847-2_18 . 29736723

[328] Seibertz F, Rapedius M, Fakuade FE, Tomsits P, Liutkute A, Cyganek L, et al. A modern automated patch-clamp approach for high throughput electrophysiology recordings in native cardiomyocytes. Commun Biol. 2022;5(1):969. Epub 20220915. doi: 10.1038/s42003-022-03871-2 ; PubMed Central PMCID: PMC9477872. 36109584 PMC9477872

[329] Gondé H, Demeules M, Hardet R, Scarpitta A, Junge M, Pinto-Espinoza C, et al. A Methodological Approach Using rAAV Vectors Encoding Nanobody-Based Biologics to Evaluate ARTC2.2 and P2X7 In Vivo. Front Immunol. 2021;12:704408. Epub 20210819. doi: 10.3389/fimmu.2021.704408 ; PubMed Central PMCID: PMC8417108. 34489954 PMC8417108

[330] Koch-Nolte F, Eichhoff A, Pinto-Espinoza C, Schwarz N, Schäfer T, Menzel S, et al. Novel biologics targeting the P2X7 ion channel. Curr Opin Pharmacol. 2019;47:110–8. Epub 20190412. doi: 10.1016/j.coph.2019.03.001 . 30986625

[331] Danquah W, Meyer-Schwesinger C, Rissiek B, Pinto C, Serracant-Prat A, Amadi M, et al. Nanobodies that block gating of the P2X7 ion channel ameliorate inflammation. Sci Transl Med. 2016;8(366):366ra162. doi: 10.1126/scitranslmed.aaf8463 . 27881823

[332] Varricchio A, Ramesh SA, Yool AJ. Novel Ion Channel Targets and Drug Delivery Tools for Controlling Glioblastoma Cell Invasiveness. Int J Mol Sci. 2021;22(21) Epub 20211102. doi: 10.3390/ijms222111909 ; PubMed Central PMCID: PMC8584308. 34769339 PMC8584308

[333] Ulrich PN, Jimenez V, Park M, Martins VP, Atwood J 3rd, Moles K, et al. Identification of contractile vacuole proteins in Trypanosoma cruzi. PLoS ONE. 2011;6(3):e18013. Epub 20110318. doi: 10.1371/journal.pone.0018013 ; PubMed Central PMCID: PMC3060929. 21437209 PMC3060929

[334] Chiurillo MA, Lander N, Vercesi AE, Docampo R. IP(3) receptor-mediated Ca(2+) release from acidocalcisomes regulates mitochondrial bioenergetics and prevents autophagy in Trypanosoma cruzi. Cell Calcium. 2020;92:102284. Epub 20200902. doi: 10.1016/j.ceca.2020.102284 . 32947181

[335] Uzcátegui NL, Figarella K, Bassarak B, Meza NW, Mukhopadhyay R, Ramirez JL, et al. Trypanosoma brucei aquaglyceroporins facilitate the uptake of arsenite and antimonite in a pH dependent way. Cell Physiol Biochem. 2013;32(4):880–8. Epub 20130927. doi: 10.1159/000354490 . 24217645

[336] Steinmann ME, Schmidt RS, Macêdo JP, Kunz Renggli C, Bütikofer P, Rentsch D, et al. Identification and characterization of the three members of the CLC family of anion transport proteins in Trypanosoma brucei. PLoS ONE. 2017;12(12):e0188219. Epub 20171215. doi: 10.1371/journal.pone.0188219 ; PubMed Central PMCID: PMC5731698. 29244877 PMC5731698

[337] Zeuthen T, Wu B, Pavlovic-Djuranovic S, Holm LM, Uzcategui NL, Duszenko M, et al. Ammonia permeability of the aquaglyceroporins from Plasmodium falciparum, Toxoplasma gondii and Trypansoma brucei. Mol Microbiol. 2006;61(6):1598–608. Epub 20060803. doi: 10.1111/j.1365-2958.2006.05325.x . 16889642

[338] Newby ZE, O’Connell J 3rd, Robles-Colmenares Y, Khademi S, Miercke LJ, Stroud RM. Crystal structure of the aquaglyceroporin PfAQP from the malarial parasite Plasmodium falciparum. Nat Struct Mol Biol. 2008;15(6):619–25. Epub 20080525. doi: 10.1038/nsmb.1431 ; PubMed Central PMCID: PMC2568999. 18500352 PMC2568999

[339] Promeneur D, Liu Y, Maciel J, Agre P, King LS, Kumar N. Aquaglyceroporin PbAQP during intraerythrocytic development of the malaria parasite Plasmodium berghei. Proc Natl Acad Sci U S A. 2007;104(7):2211–6. Epub 20070206. doi: 10.1073/pnas.0610843104 ; PubMed Central PMCID: PMC1892982. 17284593 PMC1892982

[340] Promeneur D, Mlambo G, Agre P, Coppens I. Aquaglyceroporin PbAQP is required for efficient progression through the liver stage of Plasmodium infection. Sci Rep. 2018;8(1):655. Epub 20180112. doi: 10.1038/s41598-017-18987-3 ; PubMed Central PMCID: PMC5766620. 29330527 PMC5766620

[341] Ellekvist P, Ricke CH, Litman T, Salanti A, Colding H, Zeuthen T, et al. Molecular cloning of a K(+) channel from the malaria parasite Plasmodium falciparum. Biochem Biophys Res Commun. 2004;318(2):477–484. doi: 10.1016/j.bbrc.2004.04.049 . 15120625

[342] Leippe M, Andrä J, Nickel R, Tannich E, Müller-Eberhard HJ. Amoebapores, a family of membranolytic peptides from cytoplasmic granules of Entamoeba histolytica: isolation, primary structure, and pore formation in bacterial cytoplasmic membranes. Mol Microbiol. 1994;14(5):895–904. doi: 10.1111/j.1365-2958.1994.tb01325.x . 7715451

[343] Hecht O, Van Nuland NA, Schleinkofer K, Dingley AJ, Bruhn H, Leippe M, et al. Solution structure of the pore-forming protein of Entamoeba histolytica. J Biol Chem. 2004;279(17):17834–41. Epub 20040217. doi: 10.1074/jbc.M312978200 . 14970207

[344] Bruhn H, Leippe M. Novel putative saposin-like proteins of Entamoeba histolytica different from amoebapores. Biochim Biophys Acta. 2001;1514(1):14–20. doi: 10.1016/s0005-2736(01)00345-5 . 11513801

[345] Lynch EC, Rosenberg IM, Gitler C. An ion-channel forming protein produced by Entamoeba histolytica. EMBO J. 1982;1(7):801–804. doi: 10.1002/j.1460-2075.1982.tb01250.x ; PubMed Central PMCID: PMC553112 6329705 PMC553112

[346] Zhang X, Zhang Z, Alexander D, Bracha R, Mirelman D, Stanley SL Jr. Expression of amoebapores is required for full expression of Entamoeba histolytica virulence in amebic liver abscess but is not necessary for the induction of inflammation or tissue damage in amebic colitis. Infect Immun. 2004;72(2):678–683. doi: 10.1128/IAI.72.2.678-683.2004 ; PubMed Central PMCID: PMC321641 14742508 PMC321641

